# Assembling a DNA barcode reference library for the spiders (Arachnida: Araneae) of Pakistan

**DOI:** 10.1371/journal.pone.0217086

**Published:** 2019-05-22

**Authors:** Muhammad Ashfaq, Gergin Blagoev, Hafiz Muhammad Tahir, Arif M. Khan, Muhammad Khalid Mukhtar, Saleem Akhtar, Abida Butt, Shahid Mansoor, Paul D. N. Hebert

**Affiliations:** 1 Centre for Biodiversity Genomics, University of Guelph, Guelph, ON, Canada; 2 Department of Zoology, GC University, Lahore, Pakistan; 3 Department of Biotechnology, University of Sargodha, Sargodha, Pakistan; 4 Department of Zoology, University of Sargodha, Sargodha, Pakistan; 5 Directorate of Entomology, Ayub Agricultural Research Institute, Faisalabad, Pakistan; 6 Department of Zoology, University of the Punjab, Lahore, Pakistan; 7 National Institute for Biotechnology and Genetic Engineering, Faisalabad, Pakistan; Chang Gung University, TAIWAN

## Abstract

Morphological study of 1,795 spiders from sites across Pakistan placed these specimens in 27 families and 202 putative species. COI sequences >400 bp recovered from 1,782 specimens were analyzed using neighbor-joining trees, Bayesian inference, barcode gap, and Barcode Index Numbers (BINs). Specimens of 109 morphological species were assigned to 123 BINs with ten species showing BIN splits, while 93 interim species included representatives of 98 BINs. Maximum conspecific divergences ranged from 0–5.3% while congeneric distances varied from 2.8–23.2%. Excepting one species pair (*Oxyopes azhari*–*Oxyopes oryzae*), the maximum intraspecific distance was always less than the nearest-neighbor (NN) distance. Intraspecific divergence values were not significantly correlated with geographic distance. Most (75%) BINs detected in this study were new to science, while those shared with other nations mainly derived from India. The discovery of many new, potentially endemic species and the low level of BIN overlap with other nations highlight the importance of constructing regional DNA barcode reference libraries.

## Introduction

With nearly 48,000 known species in 117 families [1], spiders are a major component of terrestrial ecosystems with important practical applications as biocontrol agents [2] and as bio-indicators [3,4]. Prior studies have documented 4,300 spider species in Europe [5] and a similar number (3,800) in the Nearctic [6]. By contrast, just 2,300 species have been reported from South Asia [7], suggesting that many species await detection in this region. Although studies on the spider fauna of Pakistan began nearly a century ago [8], work has recently intensified, but most of these studies have produced regional checklists (S1 Table). Unfortunately, these publications often employ invalid or incorrect species names or only identify specimens to a family [9], compromising their value [10–12]. It is likely that many species reported as new discoveries from Pakistan [13] await description. For example, in her dissertation research on spiders of Punjab, Parveen [13] reported the discovery of 33 new species but only one has been formally described [9]. Examination of prior taxonomic work (S1 Table) indicates that just 400 species of spiders have been documented from Pakistan. Considering the country’s diverse ecosystems [14], this count must seriously underestimate the true diversity of its fauna given the much higher numbers reported for India (1686) [15] and Iran (528) [16]. The limited knowledge of the spider fauna of Pakistan is a particular example of the barrier to our general understanding of spider biodiversity in a global context, a factor compromising both scientific progress and conservation efforts [17].

The poor documentation of spider diversity of Pakistan reflects, in part, the paucity of taxonomic specialists working on the group [18]. Moreover, spiders pose a challenge for morphological approaches because cryptic species are common [19], and sexual dimorphism is often striking [20]. DNA barcoding [21] provides an alternate approach to identifications. It employs sequence diversity in a standard gene region (COI-5′) to discriminate both morphologically cryptic species and all life stages, even for species with sexual dimorphism [22,23]. Although concerns about the use of single marker [24,25] or discordance between the barcode and other gene regions [26] have been voiced [27], the advantages of employing a single standard gene region for DNA barcoding is now very well established [28]. Fifteen years after its introduction, this approach has demonstrated its effectiveness in discriminating species in diverse groups, including spiders [29–34].

The use of DNA barcoding for specimen identification and species discovery is greatly facilitated by BOLD, the Barcode of Life Data System (http://www.boldsystems.org). This informatics platform assembles specimen metadata and sequences and provides tools to facilitate data analysis and publication [35]. It also enables species discrimination by assigning each COI sequence cluster to a Barcode Index Number (BIN) [36], which is an analogue of Operational Taxonomic Unit (OTU). Because BINs have high congruence with species recognized through morphological analysis [37–40], they are now routinely used as a species proxy [41,42]. Consequently, they have gained wide adoption [41,43] for cryptic species recognition [40,43], species discovery [44], taxonomic revisions [45], and faunal assessments [46,47]. The DNA barcode reference libraries available for diverse animal groups [48–54] are helping to identify newly collected specimens [45,54] and to speed taxonomic progress [33]. By assigning sequences from unidentified specimens to a species proxy [44], the BIN system has greatly augmented the application of barcode data in groups where taxonomic knowledge is poor. These barcode libraries are, in effect, forming the foundation for a global “DNA library of life” [55].

At present, BOLD holds 6.8 million records derived from specimens representing 587,000 BINs (accessed 13 April, 2019). This total includes 117,000 records from spiders that have been assigned to more than 10,000 BINs. Past work on spiders has had varied motivations [39,56–60], but just two prior studies have aimed to construct a comprehensive DNA barcode library for a national fauna–Canada [61] and Germany [62]. The need for similar work in other regions is evident, particularly in south Asia. For example, barcode records are only available for 73 species of spiders from India [35,63] and for 41 species from Pakistan [64–66]. The current study aimed to develop a barcode library for the spider fauna of Pakistan and investigate the spider diversity overlap with other regions using BINs. The study addresses the gap for reference data in the country by expanding DNA barcode coverage for Pakistan to 202 species.

## Materials and methods

### Ethics statement

No specific permissions were required for this study. The study did not involve endangered or protected species.

### Spider collection

**Fig 1:**
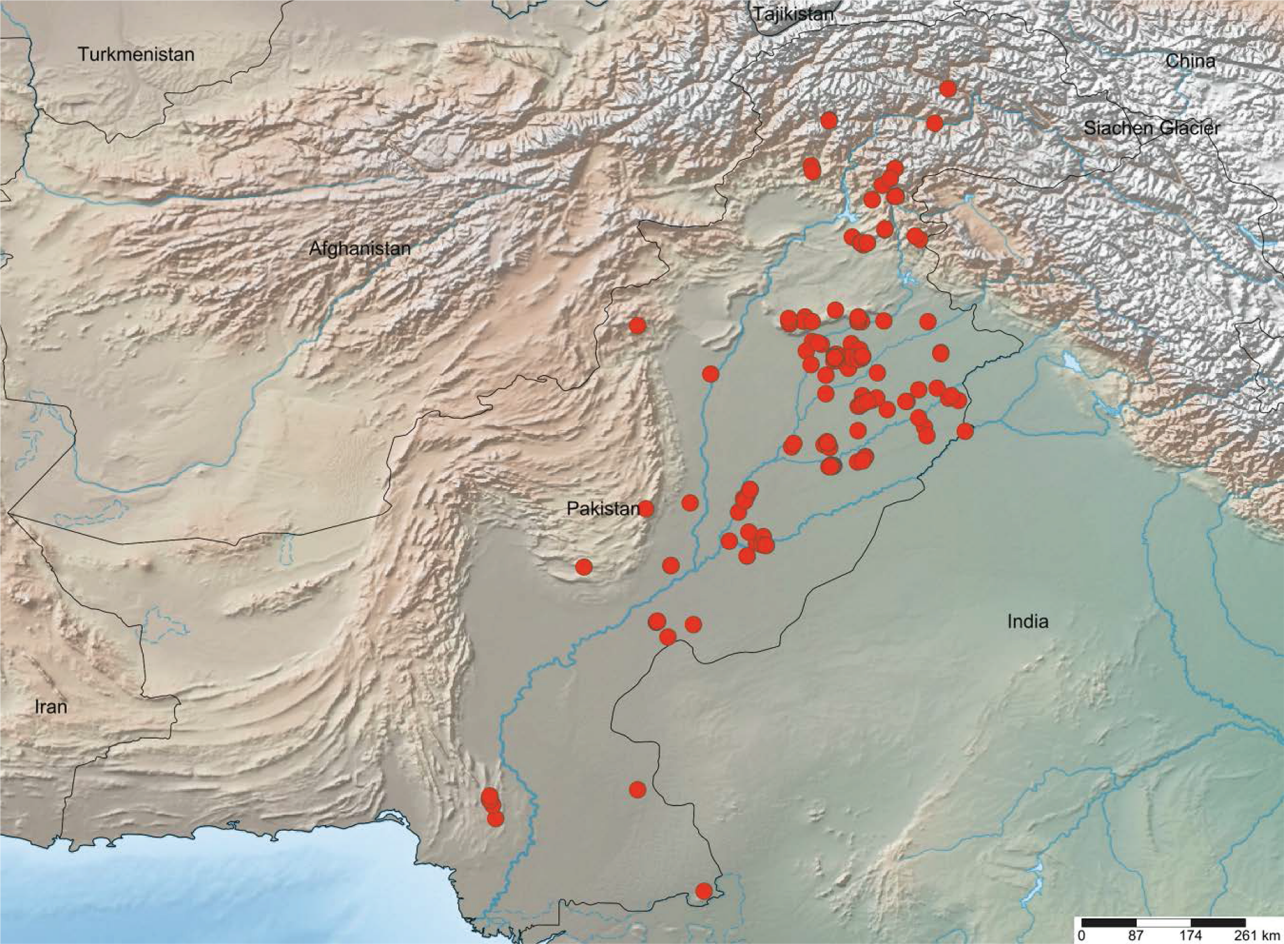
Map showing collection localities for the 1,795 spiders examined in this study. The map was developed using www.simplemappr.net. The author of SimpleMapper has waived all copyrights and no permission is needed to use. GPS coordinates (Latitude, Longitude) for the collection localities were: 24.45, 70.8; 25.488, 67.821; 25.681, 67.781; 25.756, 67.739; 25.757, 67.732; 25.759, 67.737; 25.76, 67.732; 25.801, 67.733; 25.812, 67.739; 25.9, 69.85; 28.083, 70.283; 28.261, 70.647; 28.293, 70.115; 28.304, 70.134; 28.306, 70.128; 28.308, 70.132; 28.308, 70.134; 28.309, 70.13; 28.309, 70.131; 28.309, 70.133; 29.083, 69.083; 29.103, 70.324; 29.104, 70.324; 29.105, 70.328; 29.24, 71.415; 29.242, 71.413; 29.39, 71.68; 29.393, 71.688; 29.393, 71.684; 29.394, 71.682; 29.396, 71.683; 29.401, 71.627; 29.429, 71.548; 29.454, 71.161; 29.518, 71.645; 29.584, 71.439; 29.868, 71.291; 29.9167, 69.9667; 30, 70.6; 30.026, 71.381; 30.053, 71.385; 30.065, 71.363; 30.105, 71.417; 30.189, 71.455; 30.189, 71.458; 30.189, 71.457; 30.191, 71.457; 30.516, 72.583; 30.518, 72.624; 30.519, 72.606; 30.52, 72.624; 30.522, 72.635; 30.523, 72.629; 30.525, 72.624; 30.529, 72.63; 30.531, 72.655; 30.531, 72.632; 30.533, 72.63; 30.534, 72.633; 30.534, 72.606; 30.537, 72.638; 30.538, 72.641; 30.54, 72.608; 30.585, 72.993; 30.6, 73.0667; 30.65, 73.1; 30.66, 73.1; 30.6612, 73.1086; 30.791, 72.594; 30.8, 72.05; 30.832, 72.512; 30.85, 72.083; 30.85, 72.544; 30.854, 72.538; 30.855, 72.54; 30.855, 72.539; 30.856, 72.572; 30.857, 72.542; 30.859, 72.566; 30.862, 72.56; 30.862, 72.554; 30.866, 72.555; 30.875, 72.557; 30.959, 73.984; 31.024, 74.531; 31.033, 73; 31.0833, 73.95; 31.2167, 73.8667; 31.3333, 73.4167; 31.3833, 73.0167; 31.3833, 73; 31.393, 73.027; 31.394, 73.026; 31.4167, 73.05; 31.4167, 73.0667; 31.45, 73.7; 31.45, 73.6833; 31.45, 73.1333; 31.463, 74.436; 31.4667, 73.2; 31.496, 74.294; 31.5, 73.2667; 31.532, 73.063; 31.5333, 74.3333; 31.56, 72.54; 31.6167, 73.8667; 31.64, 74.13; 31.825, 72.541; 31.8424, 70.8952; 31.86, 73.276; 31.924, 72.863; 31.965, 72.867; 31.976, 72.328; 31.986, 72.832; 32.027, 72.653; 32.034, 72.703; 32.05, 73; 32.055, 72.946; 32.059, 73.011; 32.063, 73.042; 32.0667, 72.6667; 32.0667, 72.6833; 32.067, 73.05; 32.074, 72.684; 32.077, 72.671; 32.077, 72.67; 32.078, 72.672; 32.08, 72.9; 32.081, 72.667; 32.082, 72.675; 32.083, 73.067; 32.0837, 72.6719; 32.084, 72.68; 32.088, 72.673; 32.093, 72.684; 32.1, 73.067; 32.102, 72.957; 32.109, 72.846; 32.11, 72.655; 32.119, 72.679; 32.122, 72.681; 32.125, 72.693; 32.1333, 74.1833; 32.15, 74.1833; 32.17, 72.26; 32.19, 73.025; 32.267, 72.476; 32.275, 72.904; 32.287, 72.43; 32.3054, 72.3482; 32.5333, 69.85; 32.56, 72.02; 32.59, 72.999; 32.59, 72.008; 32.59, 73.049; 32.59, 73.999; 32.591, 73.008; 32.591, 72.999; 32.5916, 72.3446; 32.592, 73.011; 32.592, 72.999; 32.593, 72.999; 32.594, 73.02; 32.594, 72.999; 32.595, 72.999; 32.5964, 72.217; 32.597, 73.041; 32.601, 73.369; 32.601, 73.038; 32.603, 73.042; 32.624, 73; 32.629, 73.009; 32.63, 73.005; 32.632, 73.013; 32.637, 73.008; 32.637, 72.008; 32.652, 73; 32.656, 73.005; 32.657, 73.004; 32.658, 73.003; 32.6581, 73.0034; 32.659, 73.008; 32.6592, 72.2433; 32.755, 72.677; 33.686, 73.076; 33.714, 73.132; 33.714, 73.133; 33.714, 73.13; 33.715, 73.132; 33.716, 73.129; 33.7167, 73.0333; 33.7167, 73.05; 33.7667, 73.8833; 33.8, 72.9167; 33.8167, 73.8167; 33.9, 73.3833; 33.9167, 73.3833; 34.333, 73.204; 34.334, 73.201; 34.38, 73.52; 34.38, 73.54; 34.385, 73.544; 34.386, 73.546; 34.386, 73.545; 34.541, 73.348; 34.543, 73.348; 34.546, 73.349; 34.638, 73.461; 34.639, 73.461; 34.639, 73.462; 34.7333, 72.35; 34.7667, 72.35; 34.776, 73.527; 34.777, 73.526; 34.778, 73.528; 34.78, 73.53; 34.78, 73.531; 34.8167, 72.3333; 35.426, 74.098; 35.461, 72.588; 35.465, 72.584; 35.4667, 72.5833; 35.478, 72.588; 35.918, 74.29; 35.918, 74.289.

From 2010 to 2016, 1,795 spiders were collected at 225 sites in Pakistan (Fig 1). Each spider was provisionally identified by collectors in Pakistan before it was sequenced for the barcode region of the mitochondrial COI gene [21]. GB subsequently validated and refined identifications by examining (including genitalic dissections) representative specimens from each barcode cluster. Generic and species assignments generally followed taxonomic publications on Asian spiders (S1 Table), but nomenclature was updated as required to follow the World Spider Catalog [1]. Collection data, a photograph, and a taxonomic assignment for each specimen are available in the public dataset, "DS-MASPD DNA barcoding spiders of Pakistan" (dx.doi.org/10.5883/DS-MASPD) on BOLD. The 1,795 specimens are held in four repositories: Centre for Biodiversity Genomics, University of Guelph, Guelph, Canada (585); National Institute for Biotechnology and Genetic Engineering, Faisalabad, Pakistan (1126); University of Sargodha, Sargodha, Pakistan (84). The location of any particular specimen is reported in the dataset.

### Molecular analysis

DNA extraction, PCR, and Sanger sequencing were performed at the Canadian Centre for DNA Barcoding (CCDB) (http://ccdb.ca/resources/) using standard protocols. A single leg was removed from each specimen with a sterile forceps and transferred into a well in a 96-well microplate pre-filled with 30 μl of 95% EtOH. DNA was subsequently extracted by tissue lysis at 56°C overnight followed by a column-based protocol [67]. PCR amplification of the COI-5′ barcode region employed the primer pair C_LepFolF and C_LepFolR (http://ccdb.ca/site/wp-content/uploads/2016/09/CCDB_PrimerSets.pdf). This primer cocktail includes equal volume of LepF1 [68] /LCO1490 [69] and LepR1 [68] /HCO2198 [69], respectively. The target COI region was amplified using 2 μL of DNA template in a 12.5 μL reaction containing standard PCR ingredients [30] employing the following PCR regime: 94°C (1 min), 5 cycles of 94°C (40 s), 45°C (40 s), 72°C (1 min); 35 cycles of 94°C (40 s), 51°C (40 s), 72°C (1 min) and final extension of 72°C (5 min). Amplicons were analyzed on a 2% agarose E-gel 96 system (Invitrogen Inc.) and were sequenced bidirectionally using the BigDye Terminator Cycle Sequencing Kit (v3.1) on an Applied Biosystems 3730XL DNA Analyzer. Sequences were assembled, aligned, and edited using CodonCode Aligner (CodonCode Corporation, USA) and validated in MEGA5 [70] to ensure they lacked a stop codon.

### Data analysis

All sequences were submitted to BOLD (DS-MASPD) where those meeting required quality criteria (>507 bp, <1% Ns, no stop codon or contamination flag) were assigned to a BIN [36]. An accumulation curve, BIN discordance, genetic distance analysis, barcode gap analysis (BGA), and geo-distance correlation were determined using analytical tools on BOLD. The Accumulation Curve plots the rise in the number of BINs with increased sampling effort making it possible to ascertain if asymptotic diversity has been reached. The BGA determines if the maximum sequence divergence within members of a species or BIN is less than the distance to its Nearest-Neighbor (NN) species or BIN, a condition required for unambiguous identification [71,72]. The geo-distance correlation ascertains the correlation between geographic distance and genetic distance in each species or BIN employing two methods. The Mantel Test [73] examines the relationship between the geographic distance (km) and genetic divergence (K2P) matrices. The second approach compares the spread of the minimum spanning tree of collection sites and maximum intra-specific divergence [61]. The relationship between geographic and intraspecific distances was analyzed for each species with at least one individual from three or more sites. The analysis included all the conspecific records public on BOLD.

A neighbor-joining (NJ) tree was generated in MEGA5 using the Kimura-2-Parameter (K2P) [74] distance model along with pairwise deletion of missing sites. Nodal support on the NJ tree was estimated by 1000 bootstrap replicates. Bayesian inference (BI) was calculated by MrBayes v3.2.0 [75] using representative sequences of the 221 BINs and employing *Phalangium opilio* (Arachnida: Opiliones) and *Galeodes* sp. (Arachnida: Solifugae) as outgroups. The data was partitioned in two ways; i) a single partition with parameters estimated across all codon positions, ii) a codon-partition in which each codon position was allowed different parameter estimates. Sequence evolution was modelled by the GTR+Γ model independently for the two partitions using the ‘‘unlink” command in MrBayes. Analyses were run for 10 million generations using four chains with sampling every 1000 generations and the BI trees were obtained using the Markov Chain Monte Carlo (MCMC) technique. Posterior probabilities were calculated from the sample points once the MCMC algorithm converged. Convergence was determined when the standard deviation of split frequencies was less than 0.022 and the PSRF (potential scale reduction factor) approached 1, and both runs converged to a stationary distribution after the burn-in stage (the first 25% of samples were discarded by default). The resultant trees were visualized in FigTree v1.4.0. The NJ and Bayesian analyses were employed to assess support for the BINs detected in this study, not to reconstruct the phylogeny of Araneae.

## Results

Coupling of the DNA sequence results with detailed morphological analysis made it possible to assign 1,574 of the 1,795 barcoded specimens to one of 109 species, but the other 221 specimens could only be placed into one of 93 interim species. Collectively, these specimens included representatives of 27 families, 113 genera, and 202 species (Table 1). Most species were only represented by a single sex, usually females. Two-thirds (1,256) of the specimens were immatures that lacked the diagnostic characters required for species assignment. However, their DNA barcodes allowed them to be linked to adults whose identification was established through morphology. Four families (Amaurobiidae, Atypidae, Ctenidae, Segestriidae), 43 genera, and 74 species identified here represent first records for Pakistan (Tables 1 and S1). As adults from 12 of the 93 interim species possessed clear morphological differences from any known species in their genus, they are likely new to science (Table 1).

**Table 1:** Species, maximum barcode divergence (K2P), nearest neighbor distance (NN), and BIN assignment of 1,795 spiders collected in Pakistan.

	No.	Taxa	*N*	K2P	NN	BINs
		**Agelenidae** C. L. Koch,1837				
	1	*Draconarius* sp. 1GAB_PAK	2	0	8.8	BOLD:AAO2052
	2	*Draconarius* sp. 2GAB_PAK	2	0	8.8	BOLD:AAO2053
*^NP^	3	*Tegenaria domestica* (Clerck, 1757)	1	N/A	19	BOLD:AAF1312
^NP^		**Amaurobiidae** Thorell,1870				
*^NS^	4	*Himalmartensus* cf. *martensi* Wang & Zhu, 2008	1	N/A	14	BOLD:ACB2928
		**Araneidae** Clerck, 1757				
^NP^	5	*Araneus affinis* Zhu, Tu & Hu, 1988	2	0.6	12	BOLD:AAV7611
	6	*Araneus mitificus* (Simon, 1886)	20	1.7	13	BOLD:AAV1598
	7	*Araniella* sp. 1GAB_PAK	4	0.8	10	BOLD:AAV1625
	8	*Argiope aemula* (Walckenaer, 1841)	8	1.4	10	BOLD:ACG0732
	9	*Argiope anasuja* Thorell, 1887	1	N/A	9.5	BOLD:ACB2926
^NP^	10	*Argiope lobata* (Pallas, 1772)	2	0.5	12	BOLD:ACI8559
^NP^	11	*Argiope pulchella* Thorell, 1881	6	0.8	9.5	BOLD:ACG0576
	12	*Argiope trifasciata* (Forsskål, 1775)	15	1.1	10	BOLD:AAQ2634
^NP^	13	*Chorizopes wulingensis* Yin, Wang & Xie, 1994	2	0.5	12	BOLD:ABX7347
^U^	14	*Cyclosa confraga* (Thorell, 1892)	8	0.8	18	BOLD:ADF2726
	15	*Cyclosa hexatuberculata* Tikader, 1982	4	0	11	BOLD:ADD8756
^NP^	16	*Cyclosa moonduensis* Tikader, 1963	9	1.1	11	BOLD:ACZ2455
	17	*Cyrtophora citricola* (Forsskål, 1775)	66	1.6	13	BOLD:AAO2032
	18	*Eriovixia excelsa* (Simon, 1889)	40	1.1	16	BOLD:AAQ0105
	19	*Gea subarmata* Thorell, 1890	1	N/A	10	BOLD:ACG0733
*^NS^	20	*Hypsosinga* cf. *alboria* Yin, Wang, Xie & Peng, 1990	4	0	10	BOLD:ABX7344
*^NP^	21	*Hypsosinga wanica* Song, Qian & Gao, 1996	17	1.6	10	BOLD:AAQ0134
	22	*Larinia phthisica* (L. Koch, 1871)	5	0.9	10	BOLD:AAO2160
	23	*Larinia* sp. 1GAB_PAK	1	N/A	11	BOLD:ABX7407
*	24	*Leviellus* sp. 1GAB_PAK	2	0	14	BOLD:AAV1590
^NP^	25	*Neoscona polyspinipes* Yin, Wang, Xie & Peng, 1990	20	0.8	4.9	BOLD:AAO1983
^NP^	26a	*Neoscona scylla* (Karsch, 1879)	13	1.9	7.8	BOLD:ACI8762
	26b	*Neoscona scylla* (Karsch, 1879)	16			BOLD:AAO1997
	27	*Neoscona* sp. 1BAG_PAK	1	N/A	7.6	BOLD:ACI2573
	28	*Neoscona* sp. 2BAG_PAK	1	N/A	9	BOLD:ADD4537
^NP^	29	*Neoscona subfusca* (C. L. Koch, 1837)	1	N/A	8.6	BOLD:AAV3851
	30	*Neoscona theisi* (Walckenaer, 1841)	160	2.5	7.6	BOLD:ACM3489
	31	*Neoscona vigilans* (Blackwall, 1865)	38	1.5	4.9	BOLD:AAO2202
	32	*Plebs himalayaensis* (Tikader, 1975)	2	0	17	BOLD:ACI8675
^NP^		**Atypidae** Thorell, 1870				
*^NS^	33	*Calommata* sp. 1GAB_PAK	1	N/A	21	BOLD:ACP9624
		**Cheiracanthiidae** Wagner, 1887				
^NP^	34	*Cheiracanthium inornatum* O. Pickard-Cambridge, 1874	5	2.3	7.4	BOLD:ACC4872
^NP^	35	*Cheiracanthium insulanum* (Thorell, 1878)	20	3.3	6.9	BOLD:AAQ0110
	36	*Cheiracanthium* sp. 1GAB_PAK	2	0.2	11	BOLD:ACA7676
	37	*Cheiracanthium* sp. 2GAB_PAK	2	1.1	4.9	BOLD:ABW2880
	38	*Cheiracanthium* sp. 3GAB_PAK	2	0.2	4.9	BOLD:AAU6055
		**Clubionidae** Wagner, 1887				
	39	*Clubiona drassodes* O. Pickard-Cambridge, 1874	28	0.9	13	BOLD:AAV1620
	40	*Clubiona filicata* O. Pickard-Cambridge, 1874	18	0.9	13	BOLD:AAV1603
	41	*Clubiona* sp. 1GAB_PAK	1	N/A	8.8	BOLD:AAV1602
	42	*Clubiona* sp. 2GAB_PAK	1	N/A	8.8	BOLD:AAO2055
		**Corinnidae** Karsch, 1880				
	43	*Castianeira* sp. 1GAB_PAK	1	N/A	16	BOLD:ACP7698
^NP^		**Ctenidae** Keyserling, 1877				
*	44	*Anahita* sp. 1GAB_PAK	1	N/A	12	BOLD:ADF5307
*	45	*Ctenus* sp. 1GAB_PAK	1	N/A	9	BOLD:AAV1591
*	46	*Ctenus* sp. 2GAB_PAK	1	N/A	9	BOLD:ABW2888
		**Filistatidae** Ausserer, 1867				
	47	*Kukulcania* sp. 1GAB_PAK	1	N/A	22	BOLD:ABX7408
		**Gnaphosidae** Pocock, 1898				
	48	*Berlandina afghana* Denis, 1958	1	N/A	14	BOLD:AAV1613
	49	*Drassodes* sp. 1GAB_PAK	1	N/A	12	BOLD:AAV1404
*^NP^	50	*Drassyllus coreanus* Paik, 1986	2	0	14	BOLD:AAV0899
	51	*Gnaphosa jodhpurensis* Tikader & Gajbe, 1977	2	1.2	15	BOLD:ACR0656
*^NP^	52	*Haplodrassus signifer* (C. L. Koch, 1839)	1	N/A	13	BOLD:ACB2432
	53	*Micaria* sp. 1GAB_PAK	1	N/A	13	BOLD:ACP3811
*	54	*Phaeocedus* sp. 1GAB_PAK	2	1.2	14	BOLD:AAV1605
*^NP^	55	*Scopoides maitraiae* (Tikader & Gajbe, 1977)	2	0	16	BOLD:ACZ1655
*^NP^	56	*Trachyzelotes kulczynskii* (Bösenberg, 1902)	1	N/A	13	BOLD:AAQ2633
^NS^	57	*Zelotes* cf. *puritanus* Chamberlin, 1922	2	0.8	12	BOLD:AAQ0137
^NP^	58	*Zelotes shantae* Tikader, 1982	1	N/A	12	BOLD:ADD7482
	59	*Zelotes* sp. 1GAB_PAK	1	N/A	12	BOLD:ACZ4032
*^NP^	60	*Zimiris diffusa* Platnick & Penney, 2004	1	N/A	14	BOLD:AAV1616
		**Hersiliidae** Thorell, 1870				
	61	*Hersilia savignyi* Lucas, 1836	16	1.1	17	BOLD:AAP4789
		**Linyphiidae** Blackwall, 1859				
	62	*Gnathonarium dentatum* (Wider, 1834)	5	0	14	BOLD:AAQ0150
*	63	*Mermessus* sp. 1GAB_PAK	1	N/A	14	BOLD:ACP3810
*^NP^	64	*Neriene emphana* (Walckenaer, 1841)	3	0.8	14	BOLD:ACI8558
		**Lycosidae** Sundevall, 1833				
*	65	*Alopecosa* sp. 1GAB_PAK	1	N/A	9.2	BOLD:AAV1615
^NS^	66	*Arctosa* cf. *serrulata* Mao & Song, 1985	1	N/A	9.4	BOLD:ACB2931
	67	*Arctosa* sp. 1GAB_PAK	1	N/A	13	BOLD:AAV1608
	68	*Draposa oakleyi* (Gravely, 1924)	19	1.6	5.8	BOLD:ABX7398
^NS^	69	*Evippa* sp. 1GAB_PAK	5	1.4	8.3	BOLD:ABX7397
	70	*Evippa* sp. 2GAB_PAK	1	N/A	8.3	BOLD:ABW2890
	71a	*Hippasa pisaurina* Pocock, 1900	16	4.1	5.8	BOLD:AAO2058
	71b	*Hippasa pisaurina* Pocock, 1900	1			BOLD:ADF3448
	72	*Hippasa* sp. 1GAB_PAK	1	N/A	5.8	BOLD:ADE8277
*	73	*Hogna* sp. 1GAB_PAK	5	0.6	10	BOLD:AAQ0158
*	74	*Hogna* sp. 2GAB_PAK	1	N/A	11	BOLD:ADF5080
	75	*Lycosa poonaensis* Tikader & Malhotra, 1980	5	0.6	10	BOLD:ABW2889
	76	*Lycosa* sp. 1GAB_PAK	1	N/A	10	BOLD:AAO2168
^E^	77	*Lycosa terrestris* Butt, Anwar & Tahir, 2006	45	0.9	4.3	BOLD:AAO2150
^NP^	78	*Pardosa mionebulosa* Yin, Peng, Xie, Bao & Wang, 1997	3	1.6	5.3	BOLD:ACZ3882
	79	*Pardosa pseudoannulata* (Bösenberg & Strand, 1906)	5	0.6	5.9	BOLD:AAO2149
	80	*Pardosa* sp. 1GAB_PAK	3	0.2	5.9	BOLD:AAO2146
	81	*Pardosa* sp. 2GAB_PAK	1	N/A	4.9	BOLD:AAO2148
	82	*Pardosa* sp. 3GAB_PAK	1	N/A	5.2	BOLD:AAV1588
	83	*Pardosa* sp. 4GAB_PAK	13	2.4	4.6	BOLD:AAO2147
	84	*Pardosa* sp. 5GAB_PAK	4	0.8	5.2	BOLD:AAV1589
^NP^	85	*Pardosa sutherlandi* (Gravely, 1924)	7	0.2	4.6	BOLD:ABX7411
^NP^	86	*Trochosa aquatica* Tanaka, 1985	17	0.6	5.9	BOLD:AAV3200
	87	*Trochosa* sp. 1GAB_PAK	3	0.3	5.9	BOLD:ADF4175
*^NP^	88	*Wadicosa fidelis* (O. Pickard-Cambridge, 1872)	75	1.9	7.2	BOLD:AAG7456
		**Oecobiidae** Blackwall, 1862				
	89	*Oecobius putus* O. Pickard-Cambridge, 1876	10	0.4	14	BOLD:AAV1624
		**Oxyopidae** Thorell, 1870				
^E^	90	*Oxyopes azhari* Butt & Beg, 2001	112	3.6	3.6	BOLD:AAO1991
^E^	91	*Oxyopes chenabensis* Mukhtar, 2017	5	0.9	6.4	BOLD:ABX7410
^NP^	92	*Oxyopes heterophthalmus* (Latreille, 1804)	8	0.3	4.9	BOLD:AAD0599
	93	*Oxyopes hindostanicus* Pocock, 1901	123	3	5.6	BOLD:AAO1990
^NP^	94	*Oxyopes macilentus* L. Koch, 1878	8	1.5	1.3	BOLD:AAF9665
^NP^	95a	*Oxyopes matiensis* Barrion & Litsinger, 1995	3	2.1	1.3	BOLD:ACX5149
	95b	*Oxyopes matiensis* Barrion & Litsinger, 1995	5			BOLD:ABX7414
^E^	96	*Oxyopes oryzae* Mushtaq & Qadar, 1999	52	1.9	3.6	BOLD:AAO1989
	97	*Oxyopes* sp. 1GAB_PAK	1	N/A	6.7	BOLD:ACZ2323
^NS^	98	*Oxyopes* sp. 2GAB_PAK	3	1.2	5.7	BOLD:ACZ4097
	99	*Oxyopes* sp. 3GAB_PAK	1	N/A	11	BOLD:ACP4193
^NP^	100	*Peucetia ranganathani* Biswas & Roy, 2005	14	0.8	11	BOLD:ACB4190
	101	*Peucetia* sp. 1GAB_PAK	1	N/A	13	BOLD:ACB4188
		**Philodromidae** Thorell, 1870				
	102	*Philodromus* sp. 1GAB_PAK	1	N/A	12	BOLD:ADD8987
	103	*Philodromus* sp. 2GAB_PAK	3	2	13	BOLD:ABX7412
*^NP^	104	*Pulchellodromus mainlingensis* (Hu & Li, 1987)	2	0	12	BOLD:ACB4189
^NS^	105	*Rhysodromus* cf. *xinjiangensis* (Tang & Song, 1987)	4	0	13	BOLD:AAO2159
^NP^	106	*Thanatus vulgaris* Simon, 1870	2	0.3	15	BOLD:AAQ0111
		**Pholcidae** C. L. Koch, 1850				
	107	*Artema* sp. 1GAB_PAK	1	N/A	19	BOLD:ABW2886
^NP^	108	*Artema transcaspica* Spassky, 1934	2	1	19	-
	109	*Crossopriza lyoni* (Blackwall, 1867)	2	0.3	16	BOLD:AAG2795
	110a	*Crossopriza maculipes* (Spassky, 1934)	4	5.3	16	BOLD:ACN4846
	110b	*Crossopriza maculipes* (Spassky, 1934)	7			BOLD:AAU5412
	110c	*Crossopriza maculipes* (Spassky, 1934)	1			BOLD:ACB2929
		**Pisauridae** Simon, 1890				
*^NP^	111	*Pisaura mirabilis* (Clerck, 1757)	4	0.5	10	BOLD:AAE4245
	112	*Pisaura* sp. 1GAB_PAK	1	N/A	12	BOLD:AAO2059
		**Salticidae** Blackwall, 1841				
^NP^	113	*Bianor albobimaculatus* (Lucas, 1846)	21	0.7	12	BOLD:AAP4728
	114	*Bianor* sp. 1GAB_PAK	1	N/A	13	BOLD:ACI8750
^NP^	115	*Epocilla sirohi* Caleb, Chatterjee, Tyagi, Kundu, Kumar, 2018	7	1.9	11	BOLD:ADD4346
	116	*Euophrys* sp. 1GAB_PAK	1	N/A	13	BOLD:ADD1307
*^NS^	117	*Evarcha* sp. 1GAB_PAK	3	0.8	9	BOLD:AAV1614
	118	*Hasarius adansoni* (Audouin, 1826)	2	0	13	BOLD:AAW0165
^NP^	119	*Hyllus dotatus* (Peckham & Peckham, 1903)	3	0.7	11	BOLD:AAV1597
^NP^	120	*Menemerus brevibulbis* (Thorell, 1887)	3	1.4	7.7	BOLD:AAO2155
	121	*Menemerus marginatus* (Kroneberg, 1875)	1	N/A	11	BOLD:AAV1611
	122	*Menemerus nigli* Wesolowska & Freudenschuss, 2012	12	1.1	7.7	BOLD:AAQ0156
*^NP^	123	*Modunda staintoni* (O. Pickard-Cambridge, 1872)	3	0.8	14	BOLD:AAV0387
^NP^	124	*Mogrus cognatus* Wesolowska & van Harten, 1994	12	1.4	8.1	BOLD:AAV1599
	125	*Mogrus* sp. 1GAB_PAK	1	N/A	8.1	BOLD:ACZ1977
	126	*Mogrus* sp. 2GAB_PAK	6	0.8	10	BOLD:AAQ2635
	127	*Myrmarachne melanocephala* MacLeay, 1839	1	N/A	6.7	BOLD:AAV1609
	128	*Myrmarachne robusta* (Peckham & Peckham, 1892)	5	1.4	6.7	BOLD:ACS0377
*^NP^	129	*Philaeus chrysops* (Poda, 1761)	1	N/A	9.8	BOLD:ACE4347
*	130	*Philaeus* sp. 1GAB_PAK	1	N/A	9.8	BOLD:AAV0574
	131	*Phintella vittata* (C. L. Koch, 1846)	11	0	9.9	BOLD:ACR1776
	132a	*Plexippus paykulli* (Audouin, 1826)	34	5	8.8	BOLD:AAO2152
	132b	*Plexippus paykulli* (Audouin, 1826)	4			BOLD:AAO2151
	132c	*Plexippus paykulli* (Audouin, 1826)	1			BOLD:ACU8433
	132d	*Plexippus paykulli* (Audouin, 1826)	1			BOLD:ABX7409
	132e	*Plexippus paykulli* (Audouin, 1826)	1			BOLD:ACZ4027
	133	*Plexippus* sp. 1GAB_PAK	2	0.2	8.8	BOLD:AAV1604
	134a	*Pseudicius admirandus* Logunov, 2007	8	1.4	9.4	BOLD:AAQ0115
	134b	*Pseudicius admirandus* Logunov, 2007	2			BOLD:ADD4534
^NP^	135	*Rhene albigera* (C. L. Koch, 1846)	1	N/A	5.9	BOLD:AAV5815
^NP^	136	*Rhene flavigera* (C. L. Koch, 1846)	4	0	5.4	BOLD:ADD7823
	137	*Rhene* sp. 1GAB_PAK	1	N/A	5.4	BOLD:ACU6737
*^NS^	138	*Sonoita* cf. *lightfooti* Peckham & Peckham, 1903	1	N/A	13	BOLD:ADD9560
	139	*Stenaelurillus arambagensis* (Biswas & Biswas, 1992)	3	0.3	11	BOLD:ABX7343
*	140	*Talavera* sp. 1GAB_PAK	1	N/A	12	BOLD:ACZ2472
	141	*Telamonia dimidiata* (Simon, 1899)	17	1.4	10	BOLD:ACG1123
	142	*Thyene imperialis* (Rossi, 1846)	56	3.5	9	BOLD:AAO2153
	143	*Thyene* sp. 1GAB_PAK	1	N/A	11	BOLD:AAV1607
*^NS^	144	*Trite* sp. 1GAB_PAK	13	0.5	11	BOLD:AAO2154
^NP^		**Segestriidae** Simon, 1893				
*	145	*Ariadna* sp. 1GAB_PAK	1	N/A	20	BOLD:AAO2054
		**Sparassidae** Bertkau, 1872				
^NP^	146	*Heteropoda maxima* Jäger, 2001	20	0.3	4.3	BOLD:ACB5077
	147a	*Heteropoda* sp. 3GAB_PAK	1	2.3	5.4	BOLD:ABW2881
	147b	*Heteropoda* sp. 3GAB_PAK	1			BOLD:AAO2057
	148	*Heteropoda* sp. 4GAB_PAK	1	N/A	4.3	BOLD:ACB5549
	149	*Olios* sp. 1GAB_PAK	1	N/A	3.9	BOLD:ADD6859
	150	*Olios* sp. 2GAB_PAK	10	0.5	3.9	BOLD:ADD7417
	151	*Olios* sp. 3GAB_PAK	4	0.3	4.1	BOLD:ACB4191
	152	*Olios* sp. 4GAB_PAK	4	1.1	7.2	BOLD:AAQ0159
	153	*Olios* sp. 5GAB_PAK	15	2.2	7.2	BOLD:AAQ0157
	154a	*Olios tener* (Thorell, 1891)	4	1.9	11	BOLD:AAQ0107
	154b	*Olios tener* (Thorell, 1891)	1			BOLD:ADK3497
	154c	*Olios tener* (Thorell, 1891)	1			BOLD:ADJ7965
	155	*Pseudopoda prompta* (O. Pickard-Cambridge, 1885)	4	0.9	13	BOLD:AAO2056
	156a	*Spariolenus tigris* Simon, 1880	1	4.1	12	BOLD:ADF5077
	156b	*Spariolenus tigris* Simon, 1880	1			BOLD:ABW2878
		**Tetragnathidae** Menge, 1866				
*^NP^	157	*Glenognatha tangi* (Zhu, Song & Zhang, 2003)	3	1.2	18	BOLD:AAQ0147
	158	*Guizygiella indica* (Tikader & Bal, 1980)	8	1.1	14	BOLD:ABX7345
	159	*Leucauge celebesiana* (Walckenaer, 1841)	7	0.2	11	BOLD:AAO2068
	160	*Leucauge decorata* (Blackwall, 1864)	30	0.5	11	BOLD:AAG8516
*	161	*Metleucauge* sp. 1GAB_PAK	1	N/A	19	BOLD:AAV1600
^NP^	162	*Tetragnatha boydi* O. Pickard-Cambridge, 1898	3	0	15	BOLD:ACB2930
^NP^	163	*Tetragnatha cavaleriei* Schenkel, 1963	2	0.5	16	BOLD:AAT8904
	164	*Tetragnatha javana* (Thorell, 1890)	43	2.8	17	BOLD:AAO2174
	165	*Tetragnatha mandibulata* Walckenaer, 1841	1	N/A	15	BOLD:AAK2567
^NP^	166	*Tetragnatha maxillosa* Thorell, 1895	4	0.3	15	BOLD:AAK2560
^NP^	167	*Tetragnatha nitens* (Audouin, 1826)	6	0.8	15	BOLD:AAD3790
	168	*Tetragnatha* sp. 1GAB_PAK	1	N/A	16	BOLD:ABW2885
		**Theraphosidae** Thorell, 1869				
*	169	*Chilobrachys* sp. 1GAB_PAK	1	N/A	4.3	BOLD:ADD5278
*	170	*Chilobrachys* sp. 2GAB_PAK	1	N/A	4.3	BOLD:AAQ0160
		**Theridiidae** Sundevall, 1833				
*^NP^	171	*Emertonella taczanowskii* (Keyserling, 1886)	1	N/A	12	BOLD:AAV1610
	172	*Enoplognatha* sp. 1GAB_PAK	1	N/A	12	BOLD:ACI8909
	173	*Enoplognatha* sp. 2GAB_PAK	1	N/A	15	BOLD:ACP4208
*	174	*Euryopis* sp. 1GAB_PAK	1	N/A	12	BOLD:AAQ0155
	175	*Latrodectus* sp. 1GAB_PAK	1	N/A	19	BOLD:AAV1732
	176	*Latrodectus* sp. 2GAB_PAK	1	N/A	16	BOLD:AAO3347
*	177	*Meotipa* sp. 1GAB_PAK	2	2	12	BOLD:AAQ0152
	178	*Phylloneta* sp. 1GAB_PAK	11	0.3	11	BOLD:AAV3043
*^NP^	179	*Steatoda cingulata* (Thorell, 1890)	2	0	14	BOLD:ABW2877
^NP^	180	*Theridion melanostictum* O. Pickard-Cambridge, 1876	1	N/A	11	BOLD:AAV1617
	181	*Theridion* sp. 1GAB_PAK	1	N/A	11	BOLD:ACB2932
	182	*Theridion* sp. 3GAB_PAK	1	N/A	12	BOLD:AAV1623
		**Thomisidae** Sundevall, 1833				
*^NP^	183	*Coriarachne melancholica* Simon, 1880	1	N/A	7.9	BOLD:ACI8639
*^NP^	184	*Ebelingia kumadai* (Ono, 1985)	3	0	12	BOLD:AAV1619
	185	*Henriksenia hilaris* (Thorell, 1877)	1	N/A	11	BOLD:AAV1618
^NP^	186	*Lysiteles kunmingensis* Song & Zhao, 1994	3	0	9.9	BOLD:ACI8899
*	187	*Misumenoides* sp. 1GAB_PAK	1	N/A	11	BOLD:AAV1594
*	188	*Misumenops* sp. 1GAB_PAK	2	1.9	11	BOLD:AAV1596
*	189	*Ozyptila* sp. 1GAB_PAK	1	N/A	11	BOLD:ADF5201
	190a	*Runcinia insecta* (L. Koch, 1875)	40	4.9	11	BOLD:AAI0997
	190b	*Runcinia insecta* (L. Koch, 1875)	2			BOLD:AAQ0108
*^NP^	191	*Tharpyna indica* Tikader & Biswas, 1979	1	N/A	12	BOLD:AAV1606
^NP^	192	*Thomisus onustus* Walckenaer, 1805	1	N/A	8.6	BOLD:AAD7031
^E^	193a	*Thomisus zaheeri* Parveen, Khan, Mushtaq, Ahmad & Rana, 2008	30	4.3	11	BOLD:AAP4819
	193b	*Thomisus zaheeri* Parveen, Khan, Mushtaq, Ahmad & Rana, 2008	1			BOLD:AAQ0153
	194	*Tmarus dostinikus* Barrion & Litsinger, 1995	13	0.2	11	BOLD:ABX7413
^NS^	195a	*Tmarus* sp. 1GAB_PAK	3	2.9	11	BOLD:ABX7346
	195b	*Tmarus* sp. 1GAB_PAK	5			BOLD:ADJ6297
	195c	*Tmarus* sp. 1GAB_PAK	4			BOLD:ADK4624
	195d	*Tmarus* sp. 1GAB_PAK	1			BOLD:ADK4625
^NP^	196	*Xysticus joyantius* Tikader, 1966	1	N/A	13	BOLD:ADF4849
	197	*Xysticus* sp. 1GAB_PAK	3	0.6	7.9	BOLD:ACI8898
	198	*Xysticus* sp. 2GAB_PAK	1	N/A	12	BOLD:ADF4647
		**Uloboridae** Thorell, 1869				
*	199	*Hyptiotes* sp. 1GAB_PAK	1	N/A	15	BOLD:AAQ2632
	200a	*Uloborus* sp. 1GAB_PAK	4	4	14	BOLD:AAW8359
	200b	*Uloborus* sp. 1GAB_PAK	1			BOLD:ABW2879
		**Zodariidae** Thorell, 1881				
*	201	*Zodarion* sp. 1GAB_PAK	1	N/A	14	BOLD:AAV1621
*	202	*Zodarion* sp. 2GAB_PAK	1	N/A	14	BOLD:ACG0983
		**Total**	**1795**			**221**

*N* = number of individuals; K2P = maximum Kimura 2-parameter distance; NN = distance to Nearest Neighbor species; BIN = Barcode Index Number; ^NP^ = new species or family to Pakistan; * = new genus to Pakistan; ^E^ = endemic species to Pakistan; ^U^ = undescribed opposite sex; ^NS^ = putative new species to science.

**Fig 2:**
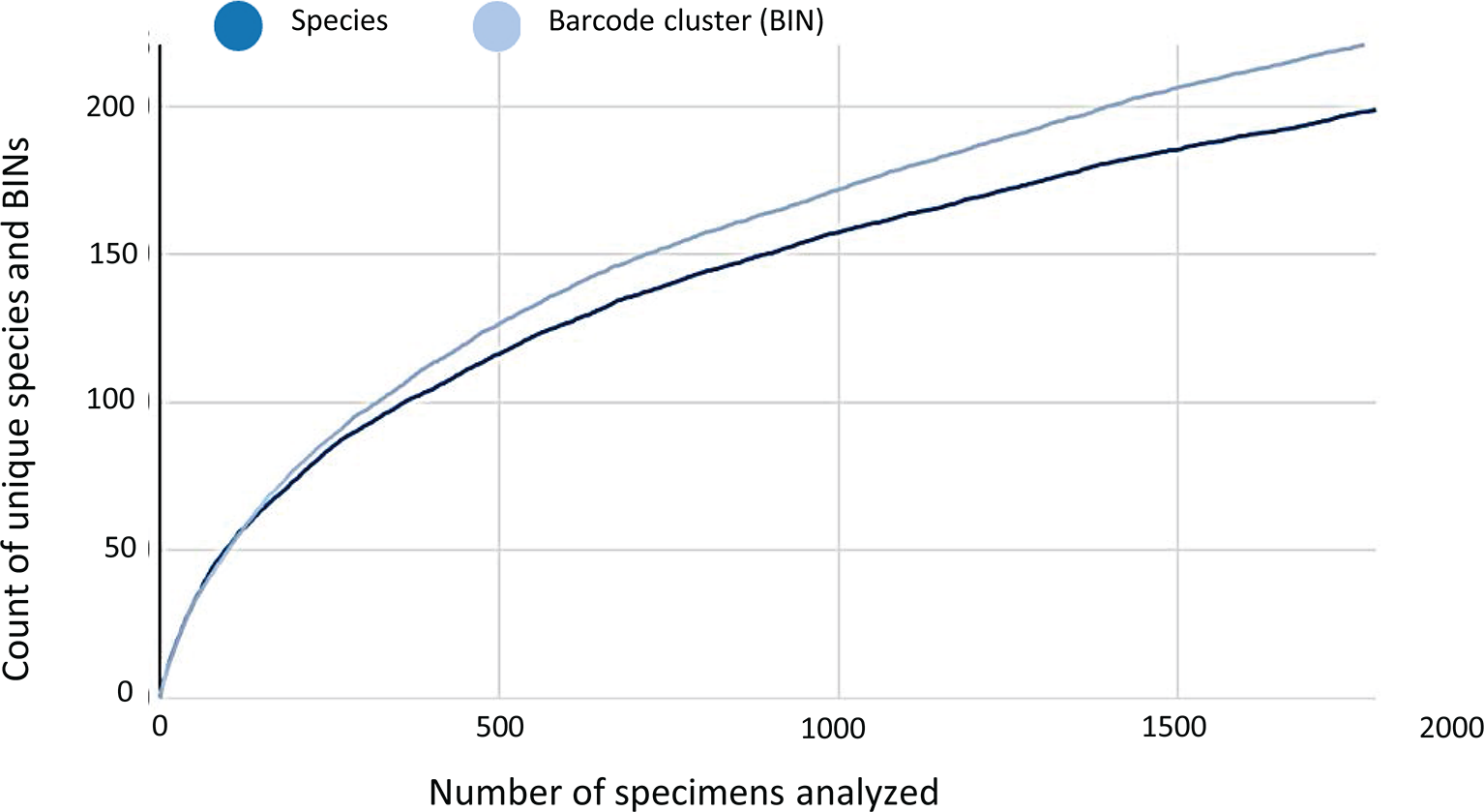
Accumulation curve for morphological species and barcode index numbers (BINs) for 1,795 spiders from Pakistan.

**Fig 3:**
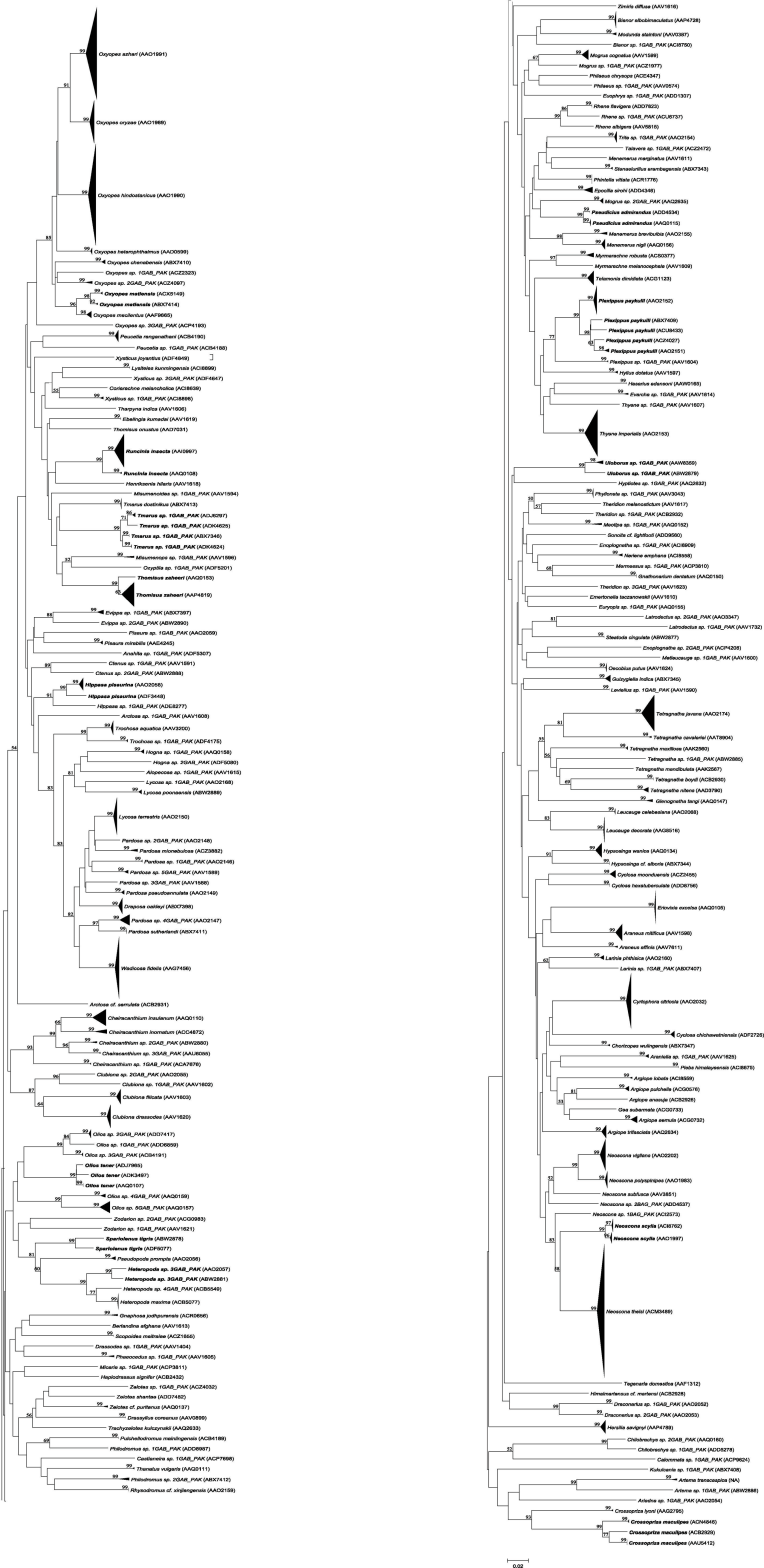
NJ analysis of spider species based on the analysis of 1,782 COI sequences. Bootstrap values (50% or higher; 1000 replicates) are shown above the branches. The scale bar shows K2P distances. The node for each species with multiple specimens is collapsed to a vertical line or triangle, with the horizontal depth indicating the level of intraspecific divergence. Species assigned to multiple BINs are indicated in **bold**. The tree is presented in two parts.

**Fig 4:**
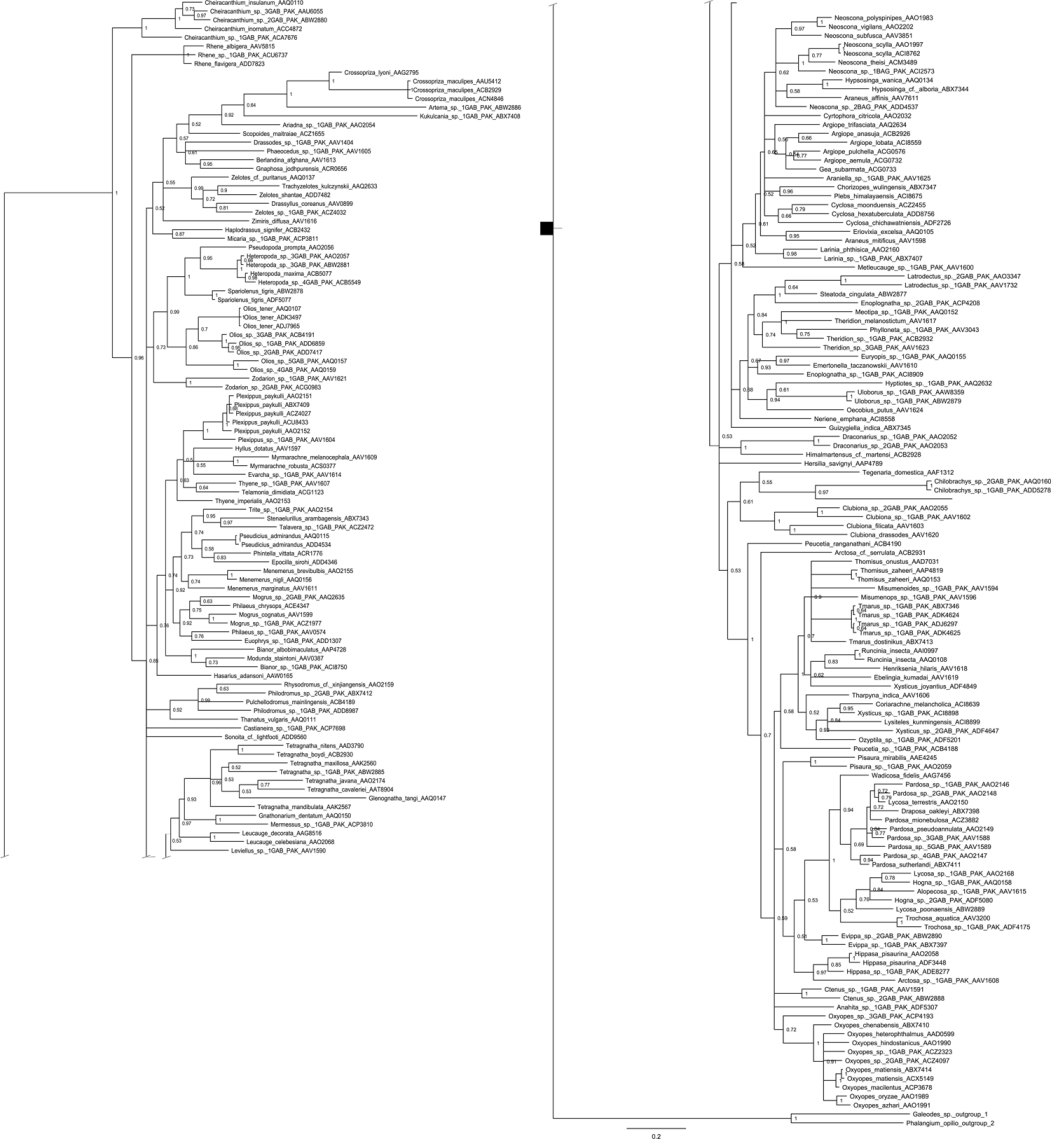
Bayesian phylogenetic analysis of spiders from Pakistan based on COI sequences. Posterior probabilities are indicated at the nodes. Taxa are followed by the BINs. *Phalangium opilio* (Arachnida: Opiliones) and *Galeodes sp*. (Arachnida: Solifugae) were employed as outgroups. Due to its large size, the tree is presented in two parts.

**Fig 5:**
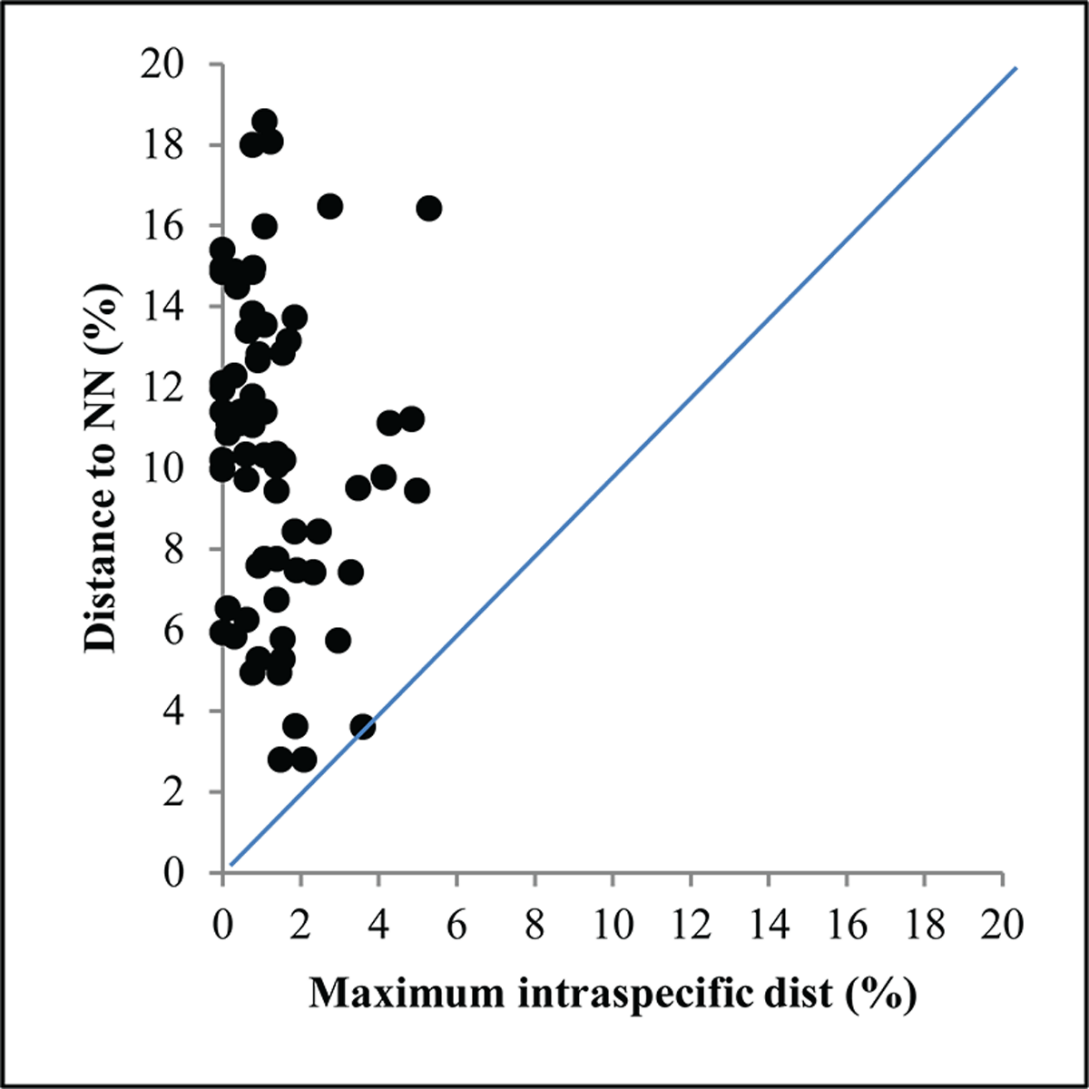
Barcode gap analysis for spider species represented by three or more records. Points that fall above the 1:1 line (blue) indicate the presence of a local barcode gap. NN = Nearest-Neighbor species.

**Fig 6:**
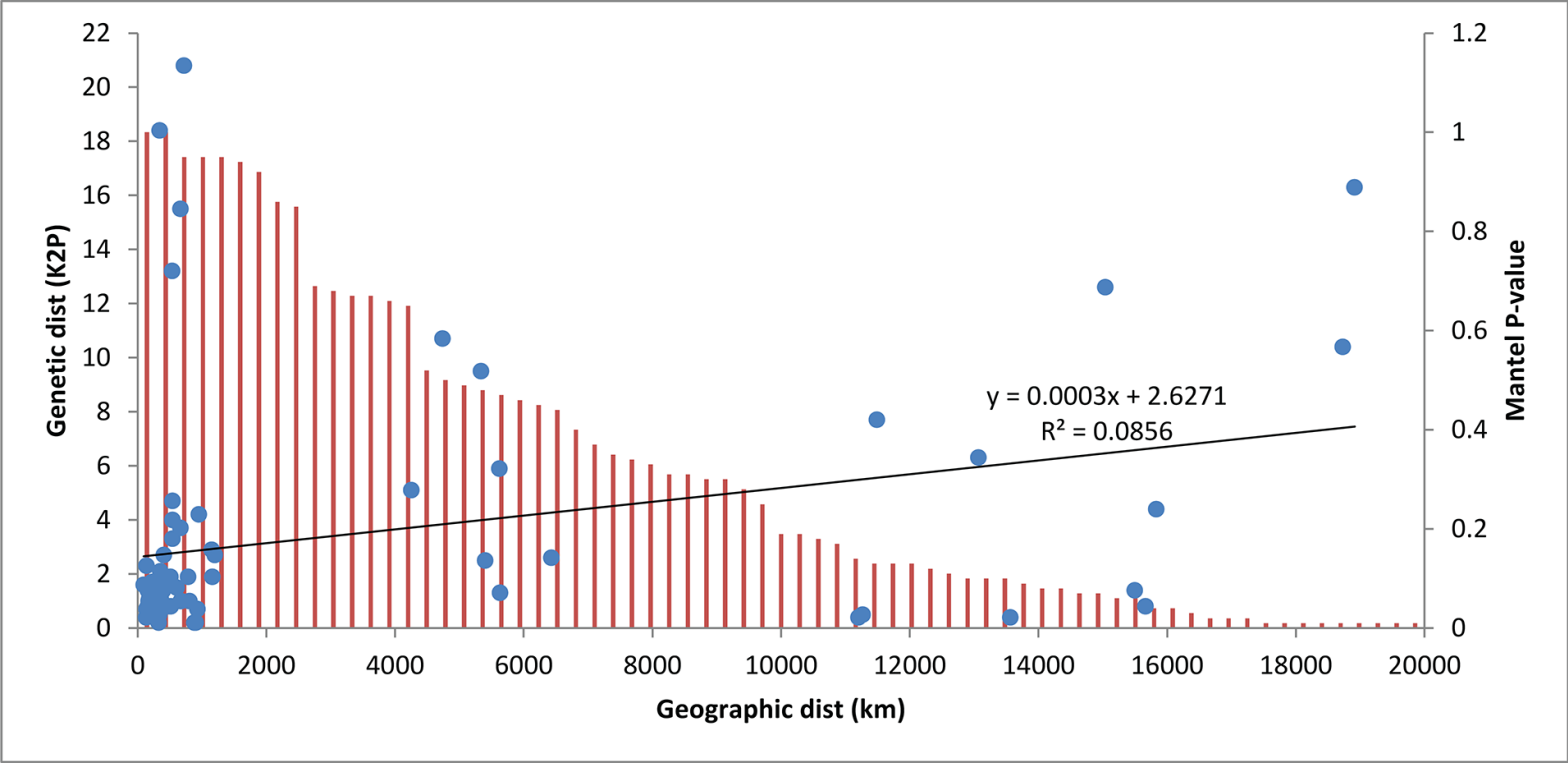
Intraspecific sequence divergence (K2P) for the COI gene (blue dots) versus geographic distance (km) for spider species from Pakistan with data from other regions. The relationship between genetic and geographic distances is indicated by a regression line. P-values for the Mantel Test are indicated by red vertical lines.

As the accumulation curve failed to approach an asymptote (Fig 2), it is certain that more species await detection. Although one species (*Artema transcaspica*) failed to qualify for a BIN assignment because its only sequence was too short, the other 108 morphological species were assigned to 123 BINs with 10 species showing a split to two or more BINs (Table 1 and Fig 3). The 93 interim species were allocated to 98 BINs with three showing BIN splits (Table 1), making the total BIN count 221 –with 94 of them singletons. NJ clustering (Fig 3) and Bayesian inference (Fig 4), supported the monophyly of all 221 BINs. Barcode distances (K2P) varied for differing taxonomic ranks with conspecific values ranging from 0.0–5.3% (mean = 0.8%), congenerics from 2.8–23.2% (mean = 8.8%), and confamilials from 4.3–26.7% (mean = 15.1%) (Table 2). Excepting 14 species, maximum intraspecific divergences did not exceed 2% in the 90 species that were represented by two or more specimens (Table 1). The barcode gap analysis showed that maximum intraspecific distance for all but one of the 90 species with two or more records was less than its NN distance (*Oxyopes azhari* was the exception, overlapping with *Oxyopes oryzae*) (Fig 5). The Mantel test was non-significant (P>0.01) for 60 of the 69 species and the regression line for all species showed a weak positive relationship (R^2^ = 0.08; y = 0.0003x + 2.62) (Fig 6).

**Table 2:** Sequence divergences (K2P) for differing levels of taxonomic affinity for the COI-5′ gene region for the spiders from Pakistan. Analysis was restricted to sequences >400 bp.

Distance class	*n*	Taxa	Comparisons	Min (%)	Mean (%)	Max (%)
Intraspecific	1702	122	44347	0	0.8	5.3
Congeners	1338	44	56792	2.8	8.8	23.2
Confamilial	1662	15	137164	4.3	15.1	26.7

**Fig 7:**
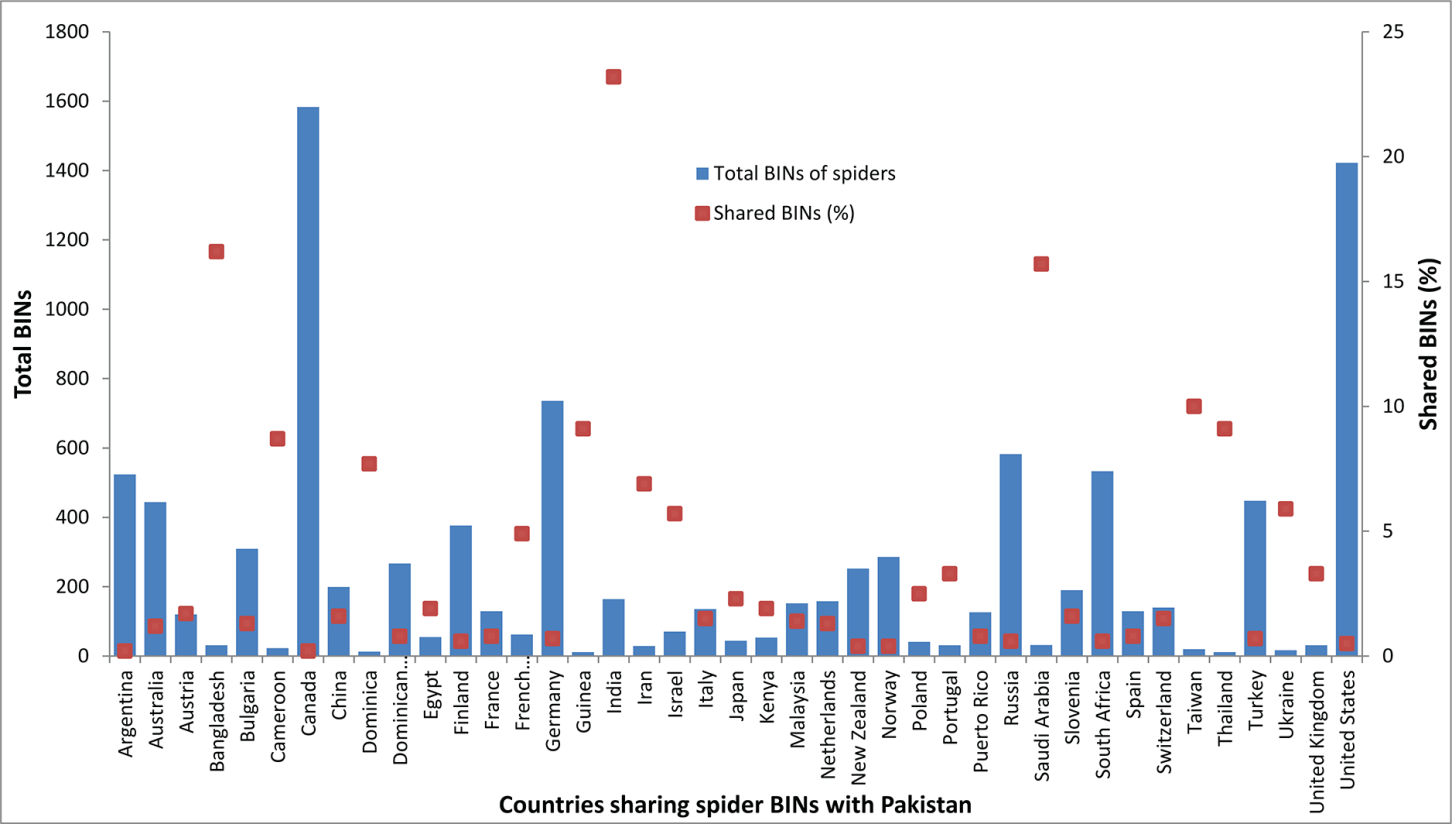
Percentage of spider BINs shared between Pakistan and 41 other nations.

The similarity between the spider fauna in Pakistan and that of other nations was calculated by examining BIN overlap. Less than a quarter (52/221) of the BINs from Pakistan were represented among the 10,229 spider BINs reported in prior studies. As expected, the highest overlap (23%) was with India, but the proportion of shared BINs was far lower for the other 43 countries (Fig 7).

## Discussion

Most prior work on the spider fauna of Pakistan has had a regional focus and only employed morphological approaches. For example, 157 species were reported from the province of Punjab [9], 56 from the district of Sargodha [76], 23 from Peshawar [11], and 13 from Buner [77]. A recent checklist for the spiders of Pakistan [10] included records for 239 species, but the present study has substantially increased this total by adding first records for 84 described species and another 93 that could not be assigned to a known taxon. Most importantly, this study generated a DNA barcode reference library for 202 species, facilitating their future identification.

Because the spider fauna of Pakistan has seen such limited study, the discovery of new species was not unexpected, and follows a pattern seen for spiders in other regions. For example, the analysis of 80 species of Salticidae from Papua New Guinea revealed 34 species and five genera new to the country [78]. Likewise, 6% of the 136 spider species recovered from the Northern Cape Province, South Africa were new [79]. This study employed a mix of methods for spider collection, including beating, sweeping, and pitfalls. The choice of sampling method impacts species detection [80] and extensive sampling is critical to generate comprehensive species coverage [81]. Although the present study involved collections at 225 sites, the resultant species accumulation curve did not reach an asymptote, indicating that many more species await detection.

The present study revealed a close correspondence (93%) between BINs and morphospecies as 188 of the 202 species were assigned to a unique BIN, reinforcing a pattern seen in other groups [37,38,40]. For example, the concordance between BINs and species was 78% in a study that examined 30,000 Canadian spiders representing 1,018 species [61] with most discordances reflecting BIN splits suggestive of overlooked species. Stronger species-BIN correspondence has been reported in several insect groups; 96% for Erebidae (Lepidoptera) from the Iberian Peninsula [38], 94% for tiger moths from Brazil [82] and 92% for beetles from central Europe [40]. However, some arthropod groups have shown relatively low level of species-BIN concordance; for example, orthopterans in Central Europe (76%) [83], waterstriders in Germany (82%) [84] and katydids in China (75%) [85]. Thirteen (6%) species in this study were assigned to two or more BINs (BIN splits), and one species (*Plexippus paykulli*) was assigned to five. BIN splits often indicate the presence of a species complex [43]. For example, 13% of 1,018 species of Canadian spiders [61], 13% of 1,541 Canadian Noctuoidea [86], 5.7% of 1,872 Finnish beetles [87], and 20% of 62 global mealybugs [88] possessed BIN splits. Although in most cases the subsequent morphological investigation has revealed overlooked species [89], other factors can cause BIN splits/mergers, such as hybridization [90], incomplete lineage sorting [83], or rapid speciation [91].

K2P divergences >2% were found in 14 of the 202 spider species from Pakistan with a maximum value of 5.3%. There was, however, no significant relationship between intraspecific divergence and the number of specimens analyzed. For example, 12 specimens of *Crossopriza maculipes* (3 BINs) showed 5.3% divergence and were assigned to three BINs while 160 specimens of *Neoscona theisi* possessed a maximum divergence of 2.5%. High COI divergence is not uncommon in spiders. For example, the maximum intraspecific divergence in 561 spider species from Germany was 10.1%, but it was below 2.5% in 95% of the cases with an arithmetic mean of 0.7% [62]. The divergence could depend on several factors such as the number of specimens analyzed, the number of localities, the geographic distance between them and the dispersal capabilities of the particular species [92,93]. With the exception of a single species (*Oxyopes azhari*), high conspecific distances did not impede the capacity of DNA barcodes to discriminate the species encountered in our study. However, species with BIN splits and high divergences are likely to represent a cryptic species complex. Preliminary morphological analyses including genitalic dissections of specimens from taxa with BIN splits in this study reinforced this conclusion.

Correlation analysis revealed only a weak relationship between the geographic range of the species examined in this study and their intraspecific divergence value. The Mantel test was significant for a few (13%) species, but species identification was not impeded as maximum intraspecific distances were nearly always less than NN distances. Similar results have been reported for Lepidoptera from Europe [94], Pakistan [32] and Central Asia [95]. Although a study that examined a single tribe, Agabini, of aquatic beetles in Europe [96] argued that regional divergences were so great as to obscure species assignments, this result is clearly not the rule [72].

Because BINs are generally an effective species proxy [41], we used them to assess faunal overlap. This work revealed that most (76%) BINs detected in this study were first records. Just 52 BINs have records from other nations and 13 of these were shared only with India. The BIN overlap with other nations was considerably lower for the spiders (24%) of Pakistan than for its Lepidoptera (42%) [42], but this difference almost certainly reflects the intensive barcode studies on the latter group. Although DNA barcoding has been used to assess regional biodiversity [41,47] and to ascertain species connections [42], the limited data availability complicates interpretation. Although further sampling will add new BINs, it is also likely to raise BIN overlap with other regions, improving our understanding of faunal overlap. Such efforts to better document local biodiversity are also certain to reveal new species as evidenced by the discovery of 93 taxa in this study that could not be assigned to a known species.

## Supporting information

S1 Table Taxonomic publications consulted for this study. (DOCX)

## References

[1] World Spider Catalog. World Spider Catalog. Version 20.0. Natural History Museum Bern, online at http://wsc.nmbe.ch, accessed on 7 January 2019. doi: 10.24436/2.

[2] Riechert SE, Lockley T. Spiders as biological control agents. Annu Rev Entomol. 1984;29: 299–320.

[3] Maelfait J-P, Hendrickx F. Spiders as bio-indicators of anthropogenic stress in natural and semi-natural habitats in Flanders (Belgium): some recent developments. In: Selden PA (ed.), Proceedings of the 17th European Colloquium of Arachnology, Edinburgh; 1998. pp. 293–300.

[4] Rybak J. Accumulation of major and trace elements in spider webs. Water Air Soil Pollut. 2015;226: 105 10.1007/s11270-015-2369-7 25821258 PMC4366562

[5] van Helsdingen PJ. The merits of a European checklist of spiders (Arachnida: Araneae). Logunov DV, Penney D, (Eds). Eur Arachnology. 2003;1: 101–106.

[6] Ubick D, Paquin P, Cushing P, Roth V. Spiders of North America: An Identification Manual, Second Edition 2nd Edition. American Arachnological Society 2017: 425 pp.

[7] Siliwal M, Molur S. Checklist of spiders (Arachnida: Araneae) of South Asia including the 2006 update of Indian spider checklist. Zoos' Print J. 2007; 22: 2551–2597.

[8] Dyal S. Fauna of Lahore. 4. -Spiders of Lahore. Bulletin of the Department of Zoology of the Panjab University. 1935; 1, i–ii, 119–252.

[9] Parveen R, Khan AA, Mushtaq S, Rana SA. A checklist of the spiders of the Punjab. Pak J Agric Sci. 2007;44: 625–626.

[10] Ghazanfar M, Hussain M, Hashim M, Fahid AM. Checklist of spider (Araneae) fauna of Pakistan: A review. J Entomol Zool Studies. 2016;4: 245–256.

[11] Perveen F, Jamal A. Checklist of spider fauna of FR Peshawar, FATA, Pakistan. Arthropods. 2012;1: 35−39.

[12] Sial N, Ruby T, Malik S, Mushtaq S. A checklist of the spiders of Cholistan and neighbouring areas. Pak J Agric Sci. 2012;49: 301–304.

[13] Parveen R. Taxonomic study on some spiders of Punjab, Pakistan. PhD Thesis. Department of Zoology and Fisheries, Agriculture University Faisalabad, Pakistan. 2003: 356 pp. http://prr.hec.gov.pk/jspui/handle/123456789//4123

[14] Baig MB, Al-Subaiee FS. Biodiversity in Pakistan: Key issues. Biodiversity 2009;10: 20–29.

[15] Keswani S, Hadole P, Rajoria A. Checklist of spiders (Arachnida: Araneae) from India-2012. Indian J Arachnol. 2012;1: 1–129.

[16] Mirshamsi O, Marusik YM, Zamani A, Moradmand M, Kashefi R. Annotated checklist of the spiders of Iran (Arachnida: Araneae). Iranian J Biosyst Fauna Iranica. 2015;1: 1–108.

[17] Borges PAV, Wunderlich J. Spider biodiversity patterns and their conservation in the Azorean archipelago, with descriptions of new species In: Systematics and Biodiversity; 2008 pp. 249–282.

[18] Drew LW. Are we losing the science of taxonomy? BioScience. 2011;61: 942–946.

[19] Zhang Y, Li S. A spider species complex revealed high cryptic diversity in South China caves. Mol Phylogenet Evol. 2014;79: 353–358. 10.1016/j.ympev.2014.05.017 24994029

[20] Hamilton CA, Formanowicz DR, Bond JE. Species delimitation and phylogeography of *Aphonopelma hentzi* (Araneae, Mygalomorphae, Theraphosidae): cryptic diversity in North American tarantulas. PLOS ONE. 2011;6: e26207 10.1371/journal.pone.0026207 22022570 PMC3192178

[21] Hebert PDN, Cywinska A, Ball SL, deWaard JR. Biological identifications through DNA barcodes. Proc Biol Sci. 2003;270: 313–321. 10.1098/rspb.2002.2218 12614582 PMC1691236

[22] Dhananjeyan KJ, Paramasivan R, Tewari SC, Rajendran R, Thenmozhi V, Leo SV, et al Molecular identification of mosquito vectors using genomic DNA isolated from eggshells, larval and pupal exuvium. Trop Biomed. 2010;27: 47–53. 20562813

[23] Iftikhar R, Ashfaq M, Rasool A, Hebert PDN. DNA barcode analysis of thrips (Thysanoptera) diversity in Pakistan reveals cryptic species complexes. PLOS ONE. 2016;11: e0146014 10.1371/journal.pone.0146014 26741134 PMC4704811

[24] Dupuis JR, Roe AD, Sperling FAH. Multi-locus species delimitation in closely related animals and fungi: one marker is not enough. Mol Ecol. 2012;21: 4422–36. 10.1111/j.1365-294X.2012.05642.x 22891635

[25] Abe TA, Spence JR, Sperling FAH. Mitochondrial introgression is restricted relative to nuclear markers in a water strider (Hemiptera: Gerridae) hybrid zone. Can J Zool. 2005;83:432–444.

[26] Pazhenkova EA, Lukhtanov VA. Nuclear genes (but not mitochondrial DNA barcodes) reveal real species: Evidence from the *Brenthis* fritillary butterflies (Lepidoptera, Nymphalidae). J Zool Syst Evol Res. 2018; 10.1111/jzs.12252

[27] Hickerson MJ, Meyer CP, Moritz C. DNA Barcoding will often fail to discover new animal species over broad parameter space. Syst Biol. 2006;55: 729–739. 10.1080/10635150600969898 17060195

[28] Stein ED, White BP, Mazor RD, Jackson JK, Battle JM, Miller PE, et al Does DNA barcoding improve performance of traditional stream bioassessment metrics? Freshw Sci. 2014;33: 302–311.

[29] Yang J, Zhang X, Zhang W, Sun J, Xie Y, Zhang Y, et al Indigenous species barcode database improves the identification of zooplankton. PLOS ONE. 2017;12: e0185697 10.1371/journal.pone.0185697 28977035 PMC5627919

[30] Hebert PDN, DeWaard JR, Zakharov EV, Prosser SW, Sones JE, McKeown JT, et al A DNA 'barcode blitz': rapid digitization and sequencing of a natural history collection. PLOS ONE. 2013;8: e68535 10.1371/journal.pone.0068535 23874660 PMC3707885

[31] Slowik J, Blagoev GA. A survey of spiders (Arachnida: Araneae) of Prince of Wales Island, Alaska; combining morphological and DNA barcode identification techniques. Insecta Mundi. 2012;251: 1–12.

[32] Blagoev GA, Nikolova NI, Sobel CN, Hebert PDN, Adamowicz SJ. Spiders (Araneae) of Churchill, Manitoba: DNA barcodes and morphology reveal high species diversity and new Canadian records. BMC Ecol. 2013;13: 44 10.1186/1472-6785-13-44 24279427 PMC4222278

[33] Cao XW, Liu J, Chen J, Zheng G, Kuntner M, Agnarsson I. Rapid dissemination of taxonomic discoveries based on DNA barcoding and morphology. Sci Rep. 2016;6: 13 10.1038/s41598-016-0006-3 27991489 PMC5171852

[34] Nadolny AA, Omelko MM, Marusik YM, Blagoev GA. A new species of spider belonging to the *Pardosa lugubris-group* (Araneae: Lycosidae) from Far East Asia. Zootaxa. 2016;4072: 263–281. 10.11646/zootaxa.4072.2.8 27395923

[35] Ratnasingham S, Hebert PDN. BOLD: The Barcode of Life Data System (http://www.barcodinglife.org). Mol Ecol Notes. 2007;7: 355–364. 10.1111/j.1471-8286.2007.01678.x 18784790 PMC1890991

[36] Ratnasingham S, Hebert PDN. A DNA-based registry for all animal species: the Barcode Index Number (BIN) system. PLOS ONE. 2013;8: e66213 10.1371/journal.pone.0066213 23861743 PMC3704603

[37] Ashfaq M, Akhtar S, Khan AM, Adamowicz SJ, Hebert PDN. DNA barcode analysis of butterfly species from Pakistan points towards regional endemism. Mol Ecol Resour. 2013;13: 832–43. 10.1111/1755-0998.12131 23789612 PMC3910150

[38] Ortiz AS, Rubio RM, Guerrero JJ, Garre MJ, Serrano J, Hebert PDN, et al Close congruence between Barcode Index Numbers (BINs) and species boundaries in the Erebidae (Lepidoptera: Noctuoidea) of the Iberian Peninsula. Biodivers Data J. 2017;5: e19840. 10.3897/BDJ.5.e19840 PMC5558050 28852323

[39] Wirta H, Varkonyi G, Rasmussen C, Kaartinen R, Schmidt NM, Hebert PDN, et al Establishing a community-wide DNA barcode library as a new tool for arctic research. Mol Ecol Resour. 2016;16: 809–822. 10.1111/1755-0998.12489 26602739

[40] Hendrich L, Morinière J, Haszprunar G, Hebert PDN, Hausmann A, Köhler F, et al A comprehensive DNA barcode database for Central European beetles with a focus on Germany: adding more than 3500 identified species to BOLD. Mol Ecol Resour. 2015;15: 795–818. 10.1111/1755-0998.12354 25469559

[41] Hebert PDN, Ratnasingham S, Zakharov EV, Telfer AC, Levesque-Beaudin V, Milton MA, et al Counting animal species with DNA barcodes: Canadian insects. Phil Trans R Soc B. 2016;371: 20150333 10.1098/rstb.2015.0333 27481785 PMC4971185

[42] Ashfaq M, Akhtar S, Rafi MA, Mansoor S, Hebert PD. Mapping global biodiversity connections with DNA barcodes: Lepidoptera of Pakistan. PLOS ONE. 2017;12: e0174749 10.1371/journal.pone.0174749 28339501 PMC5365146

[43] Ashfaq M, Hebert PDN. DNA barcodes for bio-surveillance: Regulated and economically important arthropod plant pests. Genome. 2016;59: 933–945. 10.1139/gen-2016-0024 27753511

[44] Mutanen M, Kekkonen M, Prosser SWJ, Hebert PDN, Kaila L. One species in eight: DNA barcodes from type specimens resolve a taxonomic quagmire. Mol Ecol Resour. 2015;15: 967–984. 10.1111/1755-0998.12361 25524367 PMC4964951

[45] Miller SE, Hausmann A, Hallwachs W, Janzen DH. Advancing taxonomy and bioinventories with DNA barcodes. Philos Trans Royal Soc B. 2016;371: 20150339. 10.1098/rstb.2015.0339 PMC4971191 27481791

[46] Telfer AC, Young MR, Quinn J, Perez K, Sobel CN, Sones JE, et al Biodiversity inventories in high gear: DNA barcoding facilitates a rapid biotic survey of a temperate nature reserve. Biodivers Data J. 2015;30: 2015. 10.3897/BDJ.3.e6313 PMC4568406 26379469

[47] Ashfaq M, Sabir JSM, El-Ansary HO, Perez K, Levesque-Beaudin V, Khan AM, et al Insect diversity in the Saharo-Arabian region: Revealing a little-studied fauna by DNA barcoding. PLOS ONE. 2018;13: e0199965 10.1371/journal.pone.0199965 29985924 PMC6037371

[48] Kerr KC, Stoeckle MY, Dove CJ, Weigt LA, Francis CM, Hebert PDN. Comprehensive DNA barcode coverage of North American birds. Mol Ecol Notes. 2007;7: 535–543. 10.1111/j.1471-8286.2007.01670.x 18784793 PMC2259444

[49] Hawlitschek O, Moriniere J, Dunz A, Franzen M, Rodder D, Glaw F, et al Comprehensive DNA barcoding of the herpetofauna of Germany. Mol Ecol Resour. 2016;16: 242–253. 10.1111/1755-0998.12416 25892157

[50] Dinca V, Zakharov EV, Hebert PDN, Vila R. Complete DNA barcode reference library for a country's butterfly fauna reveals high performance for temperate Europe. Proc Biol Sci. 2010;278: 347–355. 10.1098/rspb.2010.1089 20702462 PMC3013404

[51] Raupach MJ, Hendrich L, Kuchler SM, Deister F, Moriniere J, Gossner MM. Building-up of a DNA barcode library for true bugs (Insecta: Hemiptera: Heteroptera) of Germany reveals taxonomic uncertainties and surprises. PLOS ONE. 2014;9: e106940 10.1371/journal.pone.0106940 25203616 PMC4159288

[52] Moriniere J, Hendrich L, Balke M, Beermann AJ, Konig T, Hess M, et al A DNA barcode library for Germany’s mayflies, stoneflies and caddisflies (Ephemeroptera, Plecoptera and Trichoptera). Mol Ecol Resour. 2017;17: 1293–1307. 10.1111/1755-0998.12683 28449274

[53] Porco D, Chang CH, Dupont L, James S, Richard B, Decaens T. A reference library of DNA barcodes for the earthworms from Upper Normandy: Biodiversity assessment, new records, potential cases of cryptic diversity and ongoing speciation. Appl Soil Ecol. 2018;124: 362–371.

[54] Garcia-Robledo C, Kuprewicz EK, Staines CL, Kress WJ, Erwin TL. Using a comprehensive DNA barcode library to detect novel egg and larval host plant associations in a Cephaloleia rolled-leaf beetle (Coleoptera: Chrysomelidae). Biol J Linnean Soc. 2013;110: 189–198.

[55] Gall LLE, Delsuc F, Hourdez S, Lecointre G, Rasplus JY. Toward the DNA library of life. Eur J Taxon. 2017;266: 1–9.

[56] Robinson EA, Blagoev GA, Hebert PDN, Adamowicz SJ. Prospects for using DNA barcoding to identify spiders in species-rich genera. ZooKeys. 2009;16: 27–46.

[57] Blagoev GA, Dondale CD. A new species of *Alopecosa* (Araneae: Lycosidae) from Canada: a morphological description supported by DNA barcoding of 19 congeners. Zootaxa. 2014;3894: 152–160. 10.11646/zootaxa.3894.1.12 25544627

[58] Magalhaes ILF, Martins PH, Nogueira AA, Santos AJ. Finding hot singles: matching males to females in dimorphic spiders (Araneidae: *Micrathena*) using phylogenetic placement and DNA barcoding. Invertebr Syst. 2017;31: 8–36.

[59] Piacentini LN, Scioscia CL, Carbajal MN, Ott R, Brescovit AD, Ramirez MJ. A revision of the wolf spider genus *Diapontia* Keyserling, and the relationships of the subfamily Sosippinae (Araneae: Lycosidae). Arthropod Syst Phylo. 2017;75: 387–415.

[60] Ivanov V, Lee KM, Mutanen M. Mitonuclear discordance in wolf spiders: Genomic evidence for species integrity and introgression. Mol Ecol. 2018;27: 1681–1695. 10.1111/mec.14564 29575366

[61] Blagoev GA, deWaard JR, Ratnasingham S, deWaard SL, Lu L, Robertson J, et al Untangling taxonomy: a DNA barcode reference library for Canadian spiders. Mol Ecol Resour. 2016;16: 325–341. 10.1111/1755-0998.12444 26175299

[62] Astrin JJ, Hofer H, Spelda J, Holstein J, Bayer S, Hendrich L, et al Towards a DNA barcode reference database for spiders and harvestmen of Germany. PLOS ONE. 2016;11: e0162624 10.1371/journal.pone.0162624 27681175 PMC5040438

[63] Gaikwad S, Warudkar A, Shouche Y. Efficacy of DNA barcoding for the species identification of spiders from Western Ghats of India. Mitochondrial DNA Part A. 2017;28: 638–644. 10.3109/24701394.2016.1166219 27159727

[64] Tahir HM, Naseem S, Akhtar S, Ashfaq M, Butt A, Mukhtar MK. DNA barcode record of some common spiders from Punjab, Pakistan. Pak J Zool. 2016;48(1): 159–164.

[65] Naseem S, Tahir HM. Use of mitochondrial COI gene for the identification of family Salticidae and Lycosidae of spiders. Mitochondrial DNA. 2018;29: 96–101. 10.1080/24701394.2016.1248428 27841048

[66] Majeed N, Butt A, Krammer H-J, Astrin JJ. DNA barcoding in jumping spider communities of Pakistan reveals a new species (Araneae: Salticidae). ZooKeys. (In Press).

[67] Ivanova NV, deWaard JR, Hebert PDN. An inexpensive, automation-friendly protocol for recovering high quality DNA. Mol Ecol Notes. 2006;6: 998–1002.

[68] Hebert PDN, Penton EH, Burns JM, Janzen DH, Hallwachs W. Ten species in one: DNA barcoding reveals cryptic species in the neotropical skipper butterfly *Astraptes fulgerator*. Proc Natl Acad Sci USA. 2004;101: 14812–14817. 10.1073/pnas.0406166101 15465915 PMC522015

[69] Folmer O, Black M, Hoeh W, Lutz R, Vrijenhoek R. DNA primers for amplification of mitochondrial cytochrome c oxidase subunit I from diverse metazoan invertebrates. Mol Mar Biol Biotechnol. 1994;3: 294–299. 7881515

[70] Tamura K, Peterson D, Peterson N, Stecher G, Nei M, Kumar S. MEGA5: molecular evolutionary genetics analysis using maximum likelihood, evolutionary distance, and maximum parsimony methods. Mol Biol Evol. 2011;28: 2731‒2739. 10.1093/molbev/msr121 21546353 PMC3203626

[71] Meyer CP, Paulay G. DNA barcoding: Error rates based on comprehensive sampling. PLOS Biol. 2005;3: e422 10.1371/journal.pbio.0030422 16336051 PMC1287506

[72] Candek K, Kuntner M. DNA barcoding gap: reliable species identification over morphological and geographical scales. Mol Ecol Resour. 2015;15: 268–277. 10.1111/1755-0998.12304 25042335

[73] Mantel N. The detection of disease clustering and a generalized regression approach. Cancer Res. 1967;27: 209–220. 6018555

[74] Kimura M. A simple method for estimating evolutionary rates of base substitutions through comparative studies of nucleotide sequences. J Mol Evol. 1980;16: 111–120. 7463489 10.1007/BF01731581

[75] Ronquist F, Teslenko M, van der Mark P, Ayres DL, Darling A, Hohna S, et al MrBayes 3.2: efficient Bayesian phylogenetic inference and model choice across a large model space. Syst Biol. 2012;61: 539–542. 10.1093/sysbio/sys029 22357727 PMC3329765

[76] Mukhtar MK, Khan SY, Jabeen S, Tahir HM, Qadir A, Ahmad KR, et al A preliminary checklist of the spider fauna of Sargodha (Punjab), Pakistan. Pak J Zool. 2012;44: 1245–1254.

[77] Ahmad S, Akhtar N, Saeed K. Some observations on spider fauna of district Buner, Khyber Pakhtunkhwa, Pakistan. J Entomol Zool Studies. 2015;3: 47–52.

[78] Zhang J-X, Maddison WP. New euophryine jumping spiders from Papua New Guinea (Araneae: Salticidae: Euophryinae). Zootaxa. 2012;3491: 1–74.

[79] Dippenaar-Schoeman AS, Haddad CR, Lyle R, Lotz LN, Foord SH, Jocque R, et al South African National Survey of Arachnida: A checklist of the spiders (Arachnida, Araneae) of the Tswalu Kalahari Reserve in the Northern Cape province, South Africa. KOEDOE. 2018;60: a1486.

[80] King JR, Porter SD. Evaluation of sampling methods and species richness estimators for ants in upland ecosystems in Florida. Environ Entomol. 2005;34: 1566–1578.

[81] Skalak SL, Sherwin RE, Brigham RM. Sampling period, size and duration influence measures of bat species richness from acoustic surveys. Methods Ecol Evol. 2012;3: 490–502.

[82] Zenker MM, Rougerie R, Teston JA, Laguerre M, Pie MR, Freitas AVL. Fast census of moth diversity in the neotropics: A comparison of field-assigned morphospecies and DNA barcoding in tiger moths. PLOS ONE. 2016;11: e0148423 10.1371/journal.pone.0148423 26859488 PMC4747490

[83] Hawlitschek O, Moriniere J, Lehmann GUC, Lehmann AW, Kropf M, Dunz A, et al DNA barcoding of crickets, katydids and grasshoppers (Orthoptera) from Central Europe with focus on Austria, Germany and Switzerland. Mol Ecol Resour. 2017;17(5):1037–1053. 10.1111/1755-0998.12638 27863033

[84] Havemann N, Gossner MM, Hendrich L, Moriniere J, Niedringhaus R, Schafer P, et al From water striders to water bugs: the molecular diversity of aquatic Heteroptera (Gerromorpha, Nepomorpha) of Germany based on DNA barcodes. PeerJ. 2018;6: e4577 10.7717/peerj.4577 29736329 PMC5936072

[85] Zhou Z, Guo H, Han L, Chai J, Che X, Shi F. Singleton molecular species delimitation based on COI-5P barcode sequences revealed high cryptic/undescribed diversity for Chinese katydids (Orthoptera: Tettigoniidae). BMC Evol Biol. 2019;19: 79 10.1186/s12862-019-1404-5 30871464 PMC6419471

[86] Zahiri R, Lafontaine JD, Schmidt BC, DeWaard JR, Zakharov EV, Hebert PDN. A transcontinental challenge–a test of DNA barcode performance for 1,541 species of Canadian Noctuoidea (Lepidoptera). PLOS ONE. 2014;9: e92797 10.1371/journal.pone.0092797 24667847 PMC3965468

[87] Pentinsaari M, Hebert PDN, Mutanen M. Barcoding beetles: a regional survey of 1872 species reveals high identification success and unusually deep interspecific divergences. PLOS ONE. 2014;9: e108651 10.1371/journal.pone.0108651 25255319 PMC4177932

[88] Ren J-M, Ashfaq M, Hu X-N, Ma J, Liang F, Hebert PDN, et al Barcode index numbers expedite quarantine inspections and aid the interception of nonindigenous mealybugs (Pseudococcidae). Biol Invasions. 2018;20: 449–460.

[89] Landry J-F, Hebert PDN. *Plutella australiana* (Lepidoptera, Plutellidae), an overlooked diamondback moth revealed by DNA barcodes. Zookeys. 2013;327: 43–63. 10.3897/zookeys.327.5831 PMC3807746 24167421

[90] Gottsberger B, Mayer F. Behavioral sterility of hybrid males in acoustically communicating grasshoppers (Acrididae, Gomphocerinae). J Comp Physiol A Neuroethol Sens Neural Behav Physiol. 2007;193: 703–714. 10.1007/s00359-007-0225-y 17440734

[91] Hawlitschek O, Hendrich L, Espeland M, Toussaint EFA, Genner MJ, Balke M. Pleistocene climate change promoted rapid diversification of aquatic invertebrates in Southeast Australia. BMC Evolutionary Biology. 2012;12:142 10.1186/1471-2148-12-142 22873814 PMC3503846

[92] Papadopoulou A, Bergsten J, Fujisawa T, Monaghan MT, Barraclough TG, Vogler AP. Speciation and DNA barcodes: testing the effects of dispersal on the formation of discrete sequence clusters. Philos Trans R Soc Lond B Biol Sci. 2008;363: 2987–2996. 10.1098/rstb.2008.0066 18522916 PMC2607311

[93] Huemer P, Hebert PDN, Mutanen M, Wieser C, Wiesmair B, Hausmann A, et al Large geographic distance versus small DNA barcode divergence: Insights from a comparison of European to South Siberian Lepidoptera. PLOS ONE. 2018;13: e0206668 10.1371/journal.pone.0206668 30388147 PMC6214556

[94] Huemer P, Mutanen M, Sefc KM, Hebert PDN. Testing DNA barcode performance in 1000 species of European Lepidoptera: large geographic distances have small genetic impacts. PLOS ONE. 2014;9: e115774 10.1371/journal.pone.0115774 25541991 PMC4277373

[95] Lukhtanov VA, Sourakov A, Zakharov EV, Hebert PDN. DNA barcoding Central Asian butterflies: increasing geographical dimension does not significantly reduce the success of species identification. Mol Ecol Resour. 2009;9: 1302–1310. 10.1111/j.1755-0998.2009.02577.x 21564901

[96] Bergsten J, Bilton DT, Fujisawa T, Elliott M, Monaghan MT, Balke M, et al The effect of geographical scale of sampling on DNA barcoding. Syst Biol. 2012;61: 851–869. 10.1093/sysbio/sys037 22398121 PMC3417044

