# Supplementary material for: Assembling a DNA barcode reference library for the spiders (Arachnida: Araneae) of Pakistan
Source: PLoS One. 2019 May 22;14(5):e0217086. doi: 10.1371/journal.pone.0217086 (PMC6530854; doi:10.1371/journal.pone.0217086)
Supplement: S1 Table — (DOCX) [file pone.0217086.s001.docx]

**Supplementary Table 1.** Taxonomic sources.

**A.** Literature involving spiders in Pakistan.

Ali, P. A., Maddison, W. P., Zahid, M. & Butt, A. (2018). New chrysilline and aelurilline jumping spiders from Pakistan (Araneae, Salticidae). *ZooKeys*, 783, 1-15.

Ali, P. A., Zahid, M. & Butt, A. (2016). New record of spider fauna of Khyber Pakhtunkhwa Province, (Aranei; Sparassidae; Pholcidae) Pakistan. *International Journal of Biosciences*, 9(6), 247-252. doi:10.12692/ijb/9.6.247-252.

Azarkina, G. (2004). Two new species of the genus *Aelurillus* Simon, 1884 from Pakistan and Sri Lanka (Araneae: Salticidae). *Bulletin of the British Arachnological Society*, 13, 49-52.

Bauer, T., Freudenschuss, M. & Grabolle, A. (2015). *Plexippoides flavescens* (O. Pickard-Cambridge, 1872) and *Menemerus marginatus* (Kroneberg, 1875), new records for Pakistan (Aranei: Salticidae). *Arthropoda Selecta*, 24(1), 87-90.

Butt, A. & Beg, M. A. (2000). Some new species of *Marpissa* (Salticidae) spiders from Pakistan. *Pakistan Journal of Zoology*, 32, 75-79.

Butt, A. & Beg, M. A. (2001). Description of two new species of spiders of the families Clubionidae and Oxyopidae from Pakistan. *Pakistan Journal of Zoology*, 33, 35-37.

Butt, A. & Beg, M. A. (2004). New species of *Zelotus* [sic] spider (Araneae: Gnaphosidae) from Pakistan. *Pakistan Journal of Zoology*, 36, 307-312.

Butt, A. & Siraj, A. (2006). Some orb weaver spiders from Punjab, Pakistan. *Pakistan Journal of Zoology*, 38, 215-220.

Butt, A., Anwar, R. & Tahir, M. (2006). Some new species of family Lycosidae from agricultural fields of Punjab, Pakistan. *Pakistan Journal of Zoology*, 38, 185-189.

Ghafoor, A. & Alvi, Z. H. (2007). Two new species of the genus *Pardosa* (Lycosidae) from Tehsil Shorkot, district Jhang, Pakistan. *Biologia (Pakistan)*, 53, 181-186.

Ghafoor, A. & Beg, M. A. (2002a). Description of two new species of araneid spiders from Pakistan. *International Journal of Agricultural Biology*, 4, 525-527.

Ghafoor, A. & Beg, M. A. (2002b). Two new species of the genus *Gnaphosa* and *Scotophaeus* (Gnaphosidae) from Pakistan. *International Journal of Agricultural Biology*, 4, 528-530.

Ghazanfar, M., Hussain, M., Hashim, M. & fahid, A. U. M. (2016). Check list of spider (Araneae) fauna of Pakistan: A review. *Journal of Entomology and Zoology Studies*, 4(1), 245-256.

Logunov, D. V., Ballarin, F. & Marusik, Y. M. (2011). New faunistic records of the jumping and crab spiders of Karakoram, Pakistan (Aranei: Philodromidae, Salticidae and Thomisidae). *Arthropoda Selecta*, 20, 233-240.

Majeed, N., Butt, A., Krammer, H.-J. & Astrin, J. J. (In Press) DNA barcoding in jumping spider communities of Pakistan reveals a new species (Araneae: Salticidae). *ZooKeys.*

Marusik, Y. M. (2017). Redescription of *Dysdera cylindrica* (Aranei: Dysderidae) from Pakistan with notes on the distribution of the family. *Arthropoda Selecta*, 26(4), 310-314.

Marusik, Y. M. & Ballarin, F. (2011). A new species of *Draconarius* Ovtchinnikov, 1999 (Araneae, Amaurobioidea, Coelotinae) from northern Pakistan. *Zootaxa*, 2739, 27-32. doi: 10.11646/zootaxa.2739.1.2

Marusik, Y. M., Ballarin, F., Omelko, M. M. & Koponen, S. (2014). On new and interesting records of spiders from northern Pakistan and India (Aranei). *Arthropoda Selecta*, 23(4), 415-424.

Mukhtar, M. K. (2004). Taxonomic studies on the foliage spider fauna of Punjab. *Department of Zoology and Fisheries Faculty of Sciences*, *University of Agriculture*, *Faisalabad*, *Pakistan*, 244 pp. http://prr.hec.gov.pk/jspui/handle/123456789//5409

Mukhtar, M. K. (2012). Spiders of the genus *Neoscona* (Araneae: Araneidae) from Punjab, Pakistan. *Pakistan Journal of Zoology*, 44, 1711-1720.

Mukhtar, M. K. (2013a). Two new species of the genus *Oxyopes* Latreille (Arachnida: Araneae: Oxyopidae) from Punjab, Pakistan. *Pakistan Journal of Zoology*, 45, 484-488.

Mukhtar, M. K. (2013b). Description of two new species of the genus *Oxyopes* (Araneae: Oxyopidae) from Punjab, Pakistan. *Pakistan Journal of Zoology*, 45(6), 1511-1516.

Mukhtar, M. K. (2015). Two new species of the genus *Cheiracanthium* C. L. Koch (Araneae: Eutichuridae) from Punjab, Pakistan. *Pakistan Journal of Zoology*, 47(2), 467-472.

Mukhtar, M. K. (2017). Two new species of *Oxyopes* (Araneae: Oxyopidae) from Punjab, Pakistan. *Pakistan Journal of Zoology*, 49(1), 143-148. doi:10.17582/journal.pjz/2017.49.1.143.148

Mukhtar, M. K. & Mushtaq, S. (2005a). Spiders of the genus *Clubiona* (Araneae: Clubionidae) from Punjab, Pakistan. *Pakistan Journal of Zoology*, 37, 169-174.

Mukhtar, M. K. & Mushtaq, S. (2005b). Spiders of the genus *Cyclosa* (Araneae: Araneidae) from Punjab, Pakistan. *Pakistan Journal of Zoology*, 37, 199-204.

Mukhtar, M. K. & Tahir, H. M. (2013). A new species of the genus *Chorizopes* (Araneae: Araneidae) from Punjab, Pakistan. *Pakistan Journal of Zoology*, 45, 433-436.

Mukhtar, M. K., Khan, S. Y., Jabeen, S., Tahir, H. M., Qadir, A., Ahmad, K. R., Butt, A. & Arshad, M. (2012). A Preliminary Checklist of the Spider Fauna of Sargodha (Punjab), Pakistan. *Pakistan Journal of Zoology*, 44, 1245−1254.

Mushtaq, S. & Qadar, A. (1999). Three new species of the genus *Oxyopes* (Araneae: Oxyopidae) from Pakistan. *Pakistan Journal of Zoology*, 31, 255-261.

Mushtaq, S., Beg, M. A. & Waris, M. (1995). A new species and a new record for the genus *Phlegra* Simon (Araneae: Salticidae) from Pakistan. *Pakistan Journal of Zoology*, 27, 241-244.

Mushtaq, S., Beg, M. A., Waris, M. & Khan, A. A. (1995). *Myrmarachne maratha* Tikader and *Myrmarachne orientales* Tikader (Araneae: Salticidae), two new records to Pakistan. *Pakistan Journal of Zoology*, 27, 91-92.

Naseem, S. & Tahir, H. M. (2018). Use of mitochondrial COI gene for the identification of family Salticidae and Lycosidae of spiders. *Mitochondrial DNA Part A*, *2018*, 29(1), 96-101. http://dx.doi.org/10.1080/24701394.2016.1248428

Ovtchinnikov, S. V. (2006). New genus and species of spiders of the subfamily Zodariinae (Araneae, Zodariidae) from Pakistan. *Pakistan Journal of Zoology*, 40, 77-79.

Ovtchinnikov, S. V. & Inayatullah, M. (2005). Two new spider species of the genus *Draconarius* (Araneae, Amaurobiidae, Coelotinae) from Pakistan. *Vestnik Zoologii*, 39, 85-88.

Ovtchinnikov, S. V., Ahmad, B. & Inayatullah, M. (2008). Description of a new spider species of the genus *Gnaphosa* (Araneae, Gnaphosidae) from Pakistan. *Vestnik Zoologii*, 42, 81-83.

Parveen, R. (2003). Taxonomic studies on some spiders of Punjab, Pakistan. *University of Agriculture*, *Faisalabad*, *Pakistan*, 356 pp.

http://prr.hec.gov.pk/jspui/handle/123456789//4123

Parveen, R., Khan, A. A., Mushtaq, S. & Rana, S. A. (2007). A checklist of the spiders of the Punjab. *Pak. J. Agri. Sci.*, 44(4), 625-626.

Parveen, R., Khan, A. A., Mushtaq, S., Ahmad, Z. & Rana, S. A. (2008). A new species of the genus *Thomisus* Walckenaer, 1805 (Araneae: Thomisidae) from Punjab Pakistan. *Pakistan Journal of Agricultural Sciences*, 45, 119-121.

Perveen, F. & Jamal, A. (2012). Checklist of spider fauna of FR Peshawar, FATA, Pakistan. *Arthropods*, 1, 35−39.

Sameem, M. (2008). A preliminary survey of spider fauna of Malakwal, Mandi Bahauddin. *Department University of Biological Sciences University of Sargodha*, *Sargodha*, *Pakistan.* 97 pp.

Sial N., Ruby, T., Malik, S. & Mushtaq S. (2012). A checklist of the spiders of Cholistan and neighbouring areas. *Pakistan Journal of Agricultural Sciences*, 49, 301-304.

Tahir, H. M., Butt, A., Mukhtar, M. K., Bilal, M. & Khan, S. Y. (2012). Co-existence of four orb weaving spiders in the rice ecosystem. *Pakistan Journal of Zoology*, 44, 1521−1528.

Tahir, H. M., Naseem, S., Akhtar, S., Ashfaq, M., Butt A. & Mukhtar, M. K. (2016). DNA barcode record of some common spiders from Punjab, Pakistan. *Pakistan Journal of Zoology*, 48(1), 159-164.

Tahir, H. M., Nazarat, I., Naseem, S., Butt, A., Yaqoob, R., Mukhtar, M. K. & Samiullah, K. (2015). Seasonal dynamics of spiders and insect pests in Citrus Orchards of District Sargodha, Pakistan. *Pakistan Journal of Zoology*, 47, 1673−1681.

Tanasevitch, A. V. (2011). Linyphiid spiders (Araneae, Linyphiidae) from Pakistan and India. *Revue Suisse de Zoologie*, 118, 561-598.

Ursani, T. J., Soomro, N. M. (2010). Checklist of spider fauna of Sindh Province, Pakistan. *Pakistan Journal of Entomology*, 32(1), 61-73.

Wesolowska, W. & Freudenschuss, M. (2012). A new species of *Menemerus* from Pakistan and India (Araneae: Salticidae). *Genus*, 23, 449-453.

**B.** Additional literature from neighboring regions.

Bao, Y. H. & Peng, X. J. (2002). Six new species of jumping spiders (Araneae: Salticidae) from Hui-Sun Experimental Forest Station, Taiwan. *Zoological Studies*, 41, 403-411.

Bao, Y. H. & Yin, C. M. (2002). A new species of the genus *Oxyopes* from China (Araneae: Oxyopidae). *Acta Zootaxonomica Sinica*, 27, 720-722.

Barman, M. (1978). Spider genus *Oecobius* from Khasi-Jaintia hills, India (Araneae: Oecobiidae). *Science and Culture*, 44, 382-384.

Barrion, A. T. & Litsinger, J. A. (1994). Taxonomy of rice insect pests and their arthropod parasites and predators. In: Heinrichs, E. A. (ed.) Biology and Management of Rice Insects. *Wiley Eastern New Delhi*, 13-15, 283-359.

Barrion, A. T. & Litsinger, J. A. (1995). Riceland spiders of South and Southeast Asia. *CAB International Wallingford*, *UK*, xix, 700 pp.

Bayer, S. & Jäger, P. (2009). Heteropoda species from limestone caves in Laos (Araneae: Sparassidae: Heteropodinae). *Zootaxa*, 2143, 1-23.

Beatty, J. A., Berry, J. W. & Huber, B. A. (2008). The pholcid spiders of Micronesia and Polynesia (Araneae, Pholcidae). *Journal of Arachnology*, 36, 1-25.

Bhandari, R. & Gajbe, P. (2001). Description of four new species of spiders of the families Uloboridae, Philodromidae, Gnaphosidae and Lycosidae (Arachnida: Araneae) from Madhya Pradesh, India. *Records of the Zoological Survey of India*, 99, 87-93.

Biswas, B. & Biswas, K. (1992). Araneae: Spiders. *State Fauna Series 3: Fauna of West Bengal 3*, 3, 357-500.

Biswas, B. & Roy, R. (2005). Description of three new species of the genus *Peucetia* and genus *Oxyopes* (family: Oxyopidae: Araneae) from India. *Records of the Zoological Survey of India*, 105(1-2), 37-43.

Biswas, V. & Raychaudhuri, D. (2007). New record of wolf spiders (Araneae: Lycosidae) of the genus *Hippasa* Simon from Bangladesh. *Journal of the Bombay Natural History Society*, 104, 240-246.

Biswas, V. & Raychaudhuri, D. (2013a). Orb-weaving spiders of Bangladesh-I: genus *Araneus* Clerck (Araneae: Araneidae) with two new species. *Records of the Zoological Survey of India*, 113(2), 157-162.

Biswas, V. & Raychaudhuri, D. (2013b). Orb-weaving spiders of Bangladesh: genus *Neoscona* Simon (Araneae: Araneidae). *Records of the Zoological Survey of India*, 113(2), 169-188.

Biswas, V. & Raychaudhuri, D. (2015). Lynx spiders of Khulna District of Bangladesh: genus *Oxyopes* Latreille (Areaneae[sic]: Oxyopidae). *Bangladesh Journal of Zoology*, 43(2), 221-238. doi:10.3329/bjz.v43i2.27394

Bodkhe, A. K. & Vankhede, G. N. (2012). On two new species of spiders of the genus *Oxyopes* Latreille from central India (Arachnida: Araneae: Oxyopidae). *Indian Journal of Arachnology*, 1(1), 150-155.

Buchar, J. (1976). Über einige Lycosiden (Araneae) aus Nepal. *Ergebnisse des Forschungsunternehmens Nepal Himalaya*, 5, 201-227.

Buchar, J. (1978). Lycosidae aus dem Nepal-Himalaya. I. Die Gattung Acantholycosa Dahl 1908 und die *Pardosa sutherlandi* - Gruppe (Araneae: Lycosidae: Pardosinae). *Senckenbergiana Biologica*, 59, 253-265.

Cabra-García, J. J. & Brescovit, A. D. (2016). Revision and phylogenetic analysis of the orb-weaving spider genus *Glenognatha* Simon, 1887 (Araneae, Tetragnathidae). *Zootaxa*, 4069(1), 1-183. doi:10.11646/zootaxa.4069.1.1

Chen, H. M. & Zhu, M. S. (2009). One new troglophilous species of the genus *Sinopoda* (Araneae, Sparassidae) from Guizhou, China. *Acta Arachnologica*, 58, 19-21. doi:10.2476/asjaa.58.19

Chen, S. H. & Huang, W. J. (2004). A newly recorded spider of the genus *Cheiracanthium* (Araneae, Clubionidae) from Taiwan. *BioFormosa*, 39, 55-59.

Chen, Z. F. & Zhang, Z. H. (1991). Fauna of Zhejiang: Araneida. *Zhejiang Science and Technology Publishing House*, 356 pp.

Chikuni, Y. (1989). Pictorial Encyclopedia of Spiders in Japan. *Kaisei-sha Publishing Co. Tokyo*, 310 pp.

Deeleman-Reinhold, C. L. (2001). Forest spiders of South East Asia: with a revision of the sac and ground spiders (Araneae: Clubionidae, Corinnidae, Liocranidae, Gnaphosidae, Prodidomidae and Trochanterriidae [sic]). *Brill Leiden*, 591 pp.

Deeleman-Reinhold, C. L. (2009). Spiny theridiids in the Asian tropics. Systematics, notes on behaviour and species richness (Araneae: Theridiidae: Chrysso, Meotipa). *Contributions to Natural History*, 12, 403-436.

Dyal, S. (1935). Fauna of Lahore. 4.-Spiders of Lahore. *Bulletin of the Department of Zoology of the Panjab University*, 1, i-ii, 119-252.

Esyunin, S. L. & Tuneva, T. K. (2009). A review of Palaearctic lynx-spiders of the *heterophthalmus* group of the genus *Oxyopes* (Aranei, Oxyopidae). *Zoologicheskiĭ Zhurnal*, 88, 164-175. doi:10.1134/s001387380901014x

Feng, Z. Q. (1990). Spiders of China in colour. *Hunan Science and Technology Publishing House*, 256 pp.

Fourie, R., Haddad, C. R. & Jocqué, R. (2011). A revision of the purse-web spider genus *Calommata* Lucas, 1837 (Araneae, Atypidae) in the Afrotropical region. *ZooKeys*, 95, 1-28.

Gajbe, U. A. (2004a). Studies on some spiders of the family Lycosidae (Araneae: Arachnida) from Madhya Pradesh, India. *Records of the Zoological Survey of India*, 221, 1-40.

Gajbe, U. A. (2004b). Spiders of Jabalpur, Madhya Pradesh (Arachnida: Araneae). *Records of the Zoological Survey of India*, 227, 1-154.

Gajbe, U. A. (2007). Araneae: Arachnida. In: Fauna of Madhya Pradesh (including Chhattisgarh), State Fauna Series. *Zoological Survey of India*, 15(1), 419-540.

Gajbe, U. A. (2008). Fauna of India and the adjacent countries: Spider (Arachnida: Araneae: Oxyopidae). *Zoological Survey of India*, 3, 1-117.

He, S. & Hu, J. L. (2000a). A new species of the genus *Micrommata* from China (Araneae: Sparassidae). *Acta Arachnologica Sinica*, 9, 14-16.

He, S. & Hu, J. L. (2000b). A new species of the genus *Heteropoda* from Hainan Province, China (Araneae: Sparassidae). *Acta Arachnologica Sinica*, 9, 17-19.

Hu, J. L. (1984). The Chinese spiders collected from the fields and the forests. *Tianjin Press of Science and Techniques*, 482 pp.

Hu, J. L. (2001). Spiders in Qinghai-Tibet Plateau of China. *Henan Science and Technology Publishing House*, 658 pp.

Hu, J. L. & Li, A. H. (1987). The spiders collected from the fields and the forests of Xizang Autonomous Region, China. (1). *Agricultural Insects*, *Spiders*, *Plant Diseases and Weeds of Xizang*, 1, 315-392.

Hu, J. L. & Wu, W. G. (1989). Spiders from agricultural regions of Xinjiang Uygur Autonomous Region, China. *Shandong University Publishing House Jinan*, 435 pp.

Huber, B. A. (2000). New World pholcid spiders (Araneae: Pholcidae): A revision at generic level. *Bulletin of the American Museum of Natural History*, 254, 1-348.

Jäger, P. (2001). A new species of *Heteropoda* (Araneae, Sparassidae, Heteropodinae) from Laos, the largest huntsman spider? *Zoosystema*, 23, 461-465.

Jäger, P. (2006). *Martensopoda* gen. nov. from southern Indian mountain ranges, the first genus of huntsman spiders with a cymbial spur (Araneae: Sparassidae: Heteropodinae). *Zootaxa*, 1325, 335-345. doi:10.5281/zenodo.174020

Jäger, P. (2008a). Sparassidae from China 5. *Pseudopoda songi* sp. n. from Yunnan Province (Arachnida, Araneae, Sparassidae, Heteropodinae). *Senckenbergiana Biologica*, 88, 45-48.

Jäger, P. (2008b). Three new *Pseudopoda* species from northern India (Araneae, Sparassidae, Heteropodinae). *Revue Suisse de Zoologie*, 115, 515-526.

Jäger, P. (2008c). Revision of the huntsman spider genus *Heteropoda* Latreille 1804: species with exceptional male palpal conformations from Southeast Asia and Australia (Arachnida, Araneae: Sparassidae: Heteropodinae). *Senckenbergiana Biologica*, 88, 239-310.

Jäger, P. (2012a). New species of the spider genus *Olios* Walckenaer, 1837 (Araneae: Sparassidae: Sparassinae) from Laos. *Zootaxa*, 3228, 61-68. doi:10.11646/zootaxa.3228.1.3

Jäger, P. (2012b). A review on the spider genus *Argiope* Audouin 1826 with special emphasis on broken emboli in female epigynes (Araneae: Araneidae: Argiopinae). *Beiträge zur Araneologie*, 7, 272-331, 358-362.

Jäger, P. (2012c). Revision of the genus *Sinopoda* Jäger, 1999 in Laos with discovery of the first eyeless huntsman spider species (Sparassidae: Heteropodinae). *Zootaxa*, 3415, 37-57.

Jäger, P. & Kulkarni, S. (2016). An unexpected new species of the genus *Pseudopoda* (Araneae, Sparassidae, Heteropodinae) from the Western Ghats in India. *ZooKeys*, 577, 55-62. doi:10.3897/zookeys.577.7848

Jäger, P. & Praxaysombath, B. (2009). Spiders from Laos: new species and new records (Arachnida: Araneae). *Acta Arachnologica*, 58, 27-51. doi:10.2476/asjaa.58.27

Jäger, P. & Vedel, V. (2007). Sparassidae of China 4. The genus *Pseudopoda* (Araneae: Sparassidae) in Yunnan Province. *Zootaxa*, 1623, 1-38. http://dx.doi.org/10.11646/zootaxa.1623.1.1

Jäger, P., Gao, J. C. & Fei, R. I. (2002). Sparassidae in China 2. Species from the collection in Changchun (Arachnida: Araneae). *Acta Arachnologica*, 51, 23-31.

Jocqué, R. & Dippenaar-Schoeman, A. S. (2006). Spider Families of the World. *Musée Royal de l'Afrique Central Tervuren*, 336 pp.

Keswani, S. (2013). Revision of spiders from the genus *Cyclosa* (Araneae: Araneidae) with description of two new species and the first record of male of *C. moonduensis* Tikader, 1963 from India. *Indian Journal of Arachnology*, 2(1), 61-80.

Keswani, S. (2015). The genus *Dictis* L. Koch, 1872 (Araneae: Scytodidae) with description of one new species from India. *Indian Journal of Arachnology*, 4(1), 1-3.

Kim, J. P. & Cho, J. H. (2002). Spider: Natural Enemy & Resources. *Korea Research Institute of Bioscience and Biotechnology (KRIBB)*, 424 pp.

Kim, J. P. & Gwon, S. P. (2001). A revisional study of the spider family Thomisidae Sundevall, 1833 (Arachnida: Araneae) from Korea. *Korean Arachnology*, 17, 13-78.

Kim, S. T. & Lee, S. Y. (2012). Arthropoda: Arachnida: Araneae: Thomisidae. Thomisid spiders. *Invertebrate Fauna of Korea*, 21(9), 1-88.

Kim, S. T. & Lee, S. Y. (2013). Arthropoda: Arachnida: Araneae: Mimetidae, Uloboridae, Theridiosomatidae, Tetragnathidae, Nephilidae, Pisauridae, Gnaphosidae. Spiders. *Invertebrate Fauna of Korea*, 21(23), 1-183.

Kulkarni, S. & Deshpande, V. (2012). A new species of the genus *Oxyopes* Latreille (Araneae: Oxyopidae) from Sahyadri ranges of western Ghats. *Records of the Zoological Survey of India*, 112(2), 35-37.

Kulkarni, S. & Joseph, S. (2015). First record of genus *Siler* Simon, 1889 (Araneae: Salticidae) from India. *Journal of Threatened Taxa*, 7(10), 7701-7703. doi:10.11609/jott.o4266.7701-3

Lee, Y. K., Kang, S. M. & Kim, J. P. (2009). A revision of the subfamily Linyphiinae Blackwall, 1859 in Korea. *Korean Arachnology*, 25, 113-175.

Levy, G. (2007). *Calommata* (Atypidae) and new spider species (Araneae) from Israel. *Zootaxa*, 1551, 1-30. http://dx.doi.org/10.11646/zootaxa.1551.1.1

Liu, J. & Chen, J. A. (2010). A new species of the spider genus *Neriene* from southwestern China (Araneae: Linyphiidae). *Zootaxa*, 2483, 65-68.

Liu, J., Li, S. Q. & Jäger, P. (2008). New cave-dwelling huntsman spider species of the genus *Sinopoda* (Araneae: Sparassidae) from southern China. *Zootaxa*, 1857, 1-20.

Lo, Y. Y. & Lin, C. P. (2016). A new record and re-description of *Oxyopes sushilae* (Araneae, Oxyopidae) from Taiwan. *Taiwan Journal of Biodiversity*, 18(2), 137-144.

Lu, T., Wang, L. Y., Hadole, P. & Zhang, Z. S. (2016). Redescription of four wolf-spiders (Araneae: Lycosidae) from India. *Indian Journal of Arachnology*, 5, 130-142.

Majumder, S. C. & Tikader, B. K. (1991). Studies on some spiders of the family Clubionidae from India. *Records of the Zoological Survey of India*, 102, 1-175.

Malamel, J. J., Sankaran, P. M., Joseph, M. M. & Sebastian, P. A. (2015). First record of the wolf spider genus *Lysania* Thorell, 1890 from India with the description of a new species (Araneae: Lycosidae: Zoicinae). *Zootaxa*, 3904(2), 293-297. doi:10.11646/zootaxa.3904.2.8

Marusik, Y. M. & Kovblyuk, M. M. (2011). Spiders (Arachnida, Aranei) of Siberia and Russian Far East. *KMK Scientific Press Moscow*, 344 pp.

Metzner, H. (2018). Jumping spiders (Arachnida: Araneae: Salticidae) of the world. *http://www.jumping-spiders.com [accessed on 2017]*, http://www.jumping-spiders.com

Moradmand, M. (2013). The stone huntsman spider genus *Eusparassus* (Araneae: Sparassidae): systematics and zoogeography with revision of the African and Arabian species. *Zootaxa*, 3675, 1-108. doi:10.11646/zootaxa.3675.1.1

Moradmand, M., Zamani, A. & Jäger, P. (2016). On the genus *Cebrennus* Simon, 1880 in Iran with description of a new species from Iranian Central Desert (Araneae: Sparassidae). *Zootaxa*, 4121(2), 187-193. doi:10.11646/zootaxa.4121.2.9

Murphy, J. (2007). Gnaphosid genera of the world. *British Arachnological Society St Neots*, *Cambridgeshire*, 1(i-xii), 1-92, 2(i-ii), 93-605.

Namkung, J. (2002). The spiders of Korea. *Kyo-Hak Publishing Co. Seoul*, 648 pp.

Okuma, C., Kamal, N. Q., Hirashima, Y., Alam, M. Z. & Ogata, K. (1993). Illustrated Monograph of the Rice Field Spiders of Bangladesh. Institute of Postgraduate Studies in Agriculture (Salna, Gazipur, Bangladesh). *Japan International Cooperation Agency Project Publication*, 93 pp.

Ono, H. (1988). A revisional study of the spider family Thomisidae (Arachnida, Araneae) of Japan. *National Science Museum Tokyo*, 252 pp.

Ono, H. (2001). Crab spiders of the family Thomisidae from the Kingdom of Bhutan (Arachnida, Araneae). *Entomologica Basiliensis*, 23, 203-236.

Ono, H. (2009). The spiders of Japan with keys to the families and genera and illustrations of the species. *Tokai University Press Kanagawa*, 739 pp.

Ono, H., Matsuda, M. & Saito, H. (2009). Linyphiidae, Pimoidae. In: Ono, H. (ed.) The Spiders of Japan with keys to the families and genera and illustrations of the species. *Tokai University Press Kanagawa*, 253-344 pp.

Ovtsharenko, V. I., Levy, G. & Platnick, N. I. (1994). A review of the ground spider genus *Synaphosus* (Araneae, Gnaphosidae). *American Museum Novitates*, 3095, 1-27.

Paquin, P., Vink, C. & Dupérré, N. (2010). Spiders of New Zealand: annotated family key & species list. *Manaaki Whenua Press Lincoln*, *New Zealand*, 118 pp.

Patel, B. H. & Patel, H. K. (1975). On some new species of spiders of family Gnaphosidae (Araneae: Arachnida) from Gujarat, India. *Records of the Zoological Survey of India*, 68, 33-39.

Patel, B. H. & Reddy, T. S. (1993a). Two new species of the genera *Meta* C. L. Koch and *Neoscona* Simon of the family Araneidae (Arachnida: Araneae) from coastal Andhra Pradesh, India. *Records of the Zoological Survey of India*, 90, 1-6.

Patel, B. H. & Reddy, T. S. (1993b). On some new species of spiders of the genera *Hippasa* Simon, *Lycosa* Latreille, *Pardosa* Koch and *Trochosa* Koch (family: Lycosidae) from coastal Andhra Pradesh, India. *Records of the Zoological Survey of India*, 90, 121-133.

Peng, X. J. & Li, S. Q. (2003). Spiders of the genus *Plexippus* from China (Araneae: Salticidae). *Revue Suisse de Zoologie*, 110, 749-759.

Peng, X. J., Yin, C. M. & Kim, J. P. (1996). Two new species of the genus *Evippa* (Araneae: Lycosidae) from China. *Korean Arachnology*, 12(1), 71-76.

Platnick, N. I. (1976). On Asian *Prodidomus* (Araneae, Gnaphosidae). *Acta Arachnologica*, 27, 37-42.

Prajapati, D. A., Murthappa, P. S., Sankaran, P. M. & Sebastian, P. A. (2016). Two new species of *Stenaelurillus* Simon, 1886 from India (Araneae: Salticidae: Aelurillina). *Zootaxa*, 4171(2), 321-334. doi:10.11646/zootaxa.4171.2.5

Prószyński, J. (2017). Monograph of Salticidae (Araneae) of the World 1995-2015. Part II: Global Species Database of Salticidae (Araneae). *[Version October 30th 2016*, *accessed on april 2017]*,

Quan, D. & Liu, J. (2012). Two new *Rhitymna* species (Araneae: Sparassidae) from Hainan Island, China. *Zootaxa*, 3200, 61-68.

Quan, D., Zhong, Y. & Liu, J. (2014). Four *Pseudopoda* species (Araneae: Sparassidae) from southern China. *Zootaxa*, 3754(5), 555-571. doi:10.11646/zootaxa.3754.5.2

Rao, K. T., Bastawade, D. B., Javed, S. M. M. & Krishna, I. S. R. (2006). Description of two new species of spiders of the genus *Poecilotheria* Simon (Araneae: Theraphosidae) and *Tmarus* Simon (Araneae: Thomisidae) from Nallamalai Hills, eastern Ghats, Andhra Pradesh, India. *Records of the Zoological Survey of India*, 106(1), 49-54.

Saaristo, M. I. (2010). Araneae. In: Gerlach, J. & Y. Marusik (eds.) Arachnida and Myriapoda of the Seychelles islands. *Siri Scientific Press Manchester UK*, pp. 8-306.

Sankaran, P. M., Malamel, J. J., Joseph, M. M. & Sebastian, P. A. (2015). An updated review of the genus *Martensopoda* Jäger, 2006 (Araneae: Sparassidae: Heteropodinae). *Zootaxa*, 3937(3), 577-590. doi:10.11646/zootaxa.3937.3.9

Sen, S., Dhali, D. C., Saha, S. & Raychaudhuri, D. (2015). Spiders (Araneae: Arachnida) of Reserve Forests of Dooars: Gorumara National Park, Chapramari Wildlife Sanctuary and Mahananda Wildlife Sanctuary. *World Scientific News*, 20, 1-339.

Sethi, V. D. & Tikader, B. K. (1988). Studies on some giant crab spiders of the family Heteropodidae from India. *Records of the Zoological Survey of India*, 93, 1-94.

Song, D. X. (1991). On lynx spiders of the genus *Oxyopes* (Araneae: Oxyopidae) from China. *Sinozoologia*, 8, 169-181.

Song, D. X. & Chai, J. Y. (1990). Notes of some species of the family Thomisidae (Arachnida: Araneae) from Wuling Shan area. *From Water onto Land. C.S.S.A.R. Beijing*, pp. 364-374.

Song, D. X. & Li, S. Q. (1997). Spiders of Wuling Mountains area. In: Song, D. X. (ed.) Invertebrates of Wuling Mountains Area, Southwestern China. *Science Press Beijing*, pp. 400-448.

Song, D. X. & Zhu, M. S. (1997). Fauna Sinica: Arachnida: Araneae: Thomisidae, Philodromidae. *Science Press Beijing*, viii, 259 pp.

Song, D. X., Zhu, M. S. & Chen, J. (1999). The spiders of China. *Hebei University of Science and Techology Publishing House Shijiazhuang*, 640 pp.

Song, D. X., Zhu, M. S. & Chen, J. (2001). The Fauna of Hebei, China: Araneae. *Hebei University of Science and Techology Publishing House Shijiazhuang*, 510 pp.

Song, D. X., Zhu, M. S. & Li, S. Q. (1993). Arachnida: Araneae. In: Huang, C. M. (ed.) Animals of Longqi Mountai. *China Forestry Publishing House Beijing*, 852-890 pp.

Song, D. X., Zhu, M. S. & Zhang, F. (2004). Fauna Sinica: Invertebrata Vol. 39: Arachnida: Araneae: Gnaphosidae. *Science Press Beijing*, 362 pp.

Spassky, S. A. (1934). Araneae palaearcticae novae. Fam. Pholcidae. *Bulletin du Muséum National d'Histoire Naturelle de Paris (2)*, 6, 361-372.

Sun, C. K., Li, X. H. & Zhang, F. (2011). A new species of the genus *Olios* (Araneae: Sparassidae) from Hainan Island, China. *Acta Arachnologica Sinica*, 20, 88-90.

Tanaka, H. (2009). Lycosidae. In: Ono, H. (ed.) The Spiders of Japan with keys to the families and genera and illustrations of the species. *Tokai University Press Kanagawa*, 222-248 pp.

Tang, G. & Li, S. Q. (2009). The crab spiders of the genus *Tmarus* from Xishuangbanna, Yunnan, China (Araneae: Thomisidae). *Zootaxa*, 2223, 48-68. doi:10.11646/zootaxa.2223.1.3

Tang, G. & Li, S. Q. (2010a). Crab spiders from Hainan Island, China (Araneae, Thomisidae). *Zootaxa*, 2369, 1-68.

Tang, G. & Li, S. Q. (2010b). Crab spiders from Xishuangbanna, Yunnan Province, China (Araneae, Thomisidae). *Zootaxa*, 2703, 1-105.

Tang, G. & Li, S. Q. (2012). Lynx spiders from Xishuangbanna, Yunnan, China (Araneae: Oxyopidae). *Zootaxa*, 3362, 1-42.

Tang, G., Peng, X. J., Griswold, C., Ubick, D. & Yin, C. M. (2008). Four crab spiders of the family Thomisidae (Araneae, Thomisidae) from Yunnan, China. *Acta Zootaxonomica Sinica*, 33, 241-247.

Tang, G., Yin, C. M., Peng, X. J., Ubick, D. & Griswold, C. (2007). Five crab spiders of the genus *Lysiteles* from Yunnan Province, China (Araneae: Thomisidae). *Zootaxa*, 1480, 57-68. http://dx.doi.org/10.11646/zootaxa.1480.1.2

Tang, G., Yin, C. M., Peng, X. J., Ubick, D. & Griswold, C. (2008). The crab spiders of the genus *Lysiteles* from Yunnan Province, China (Araneae: Thomisidae). *Zootaxa*, 1742, 1-41. http://dx.doi.org/10.11646/zootaxa.1480.1.2

Tang, G., Yin, C. M., Ubick, D. & Peng, X. J. (2008). Two new species of the crab spider genus *Talaus* (Araneae: Thomisidae) from Yunnan province, China. *Zootaxa*, 1815, 62-68.

Tanikawa, A. (2009). Hersiliidae. Nephilidae, Tetragnathidae, Araneidae. In: Ono, H. (ed.) The Spiders of Japan with keys to the families and genera and illustrations of the species. *Tokai University Press Kanagawa*, 149, 403-463.

Tanikawa, A. (2013). Two new species of the genus *Cyrtarachne* (Araneae: Araneidae) from Japan hitherto identified as *C. inaequalis*. *Acta Arachnologica*, 62(2), 95-101. doi:10.2476/asjaa.62.95

Tikader, B. K. (1980). Thomisidae (Crab-spiders). *The Fauna of India (Araneae) 1*, 1, 1-247.

Tikader, B. K. (1982a). Family Araneidae (=Argiopidae), typical orbweavers. *Fauna India (Araneae) 2*, 2, 1-293.

Tikader, B. K. (1982b). Family Gnaphosidae. *Fauna India (Araneae) 3*, 3, 295-536.

Tikader, B. K. & Bal, A. (1981). Studies on some orb-weaving spiders of the genera *Neoscona* Simon and *Araneus* Clerck of the family Araneidae (=Argiopidae) from India. *Records of the Zoological Survey of India*, 24, 1-60.

Tikader, B. K. & Biswas, B. (1981). Spider fauna of Calcutta and vicinity: Part-I. *Records of the Zoological Survey of India*, 30, 1-149.

Tikader, B. K. & Malhotra, M.S. (1980). Lycosidae (Wolf-spiders). *The Fauna of India (Araneae) 1*, 1, 248-447.

Tikader, B. K. & Sethi, V. D. (1990). Studies of some giant crab spiders of the family Heteropodidae from India. Part II. *Records of the Zoological Survey of India*, 87, 165-186.

Tong, Y. F. (2013). Haplogynae Spiders from Hainan, China. *Ke xue chu ban she Beijing*, vi, 96 pp, 81 pl.

Tu, L. H. & Li, S. Q. (2006). Three new and four newly recorded species of Linyphiinae and Micronetinae spiders (Araneae: Linyphiidae) from northern Vietnam. *Raffles Bulletin of Zoology*, 54, 103-117.

Vedel, V. & Jäger, P. (2005). Sparassidae of China 3. First record of the genus *Bhutaniella* in China (Araneae: Sparassidae) with descriptions of two new species. *Acta Arachnologica*, 54, 41-43.

Wang, X. G. & Xi, G. S. (1998). A new species of the genus *Tmarus* from Shaanxi Province, China (Araneae: Thomisidae). *Acta Arachnologica Sinica*, 7, 33-35.

Wanless, F. R. (1985). A revision of the spider genera *Holcolaetis* and *Sonoita* (Araneae: Salticidae). *Bulletin of the British Museum of Natural History (Zool.)*, 48, 249-278.

Wesolowska, W. & Harten, A. van (1994). The jumping spiders (Salticidae, Araneae) of Yemen. *Yemeni-German Plant Protection Project Sana'a*, 86 pp.

World Spider Catalog (2017). World Spider Catalog. Natural History Museum Bern, Bern. *http://wsc.nmbe.ch [version 18.0*, *accessed on March 2017]*, http://wsc.nmbe.ch

Xie, L. P. & Kim, J. P. (1996). Three new species of the genus *Oxyopes* from China (Araneae: Oxyopidae). *Korean Arachnology*, 12(2), 33-40.

Yang, X. F. & Chai, B. Q. (1998). A study on five wolf spiders of the group *Pardosa nebulosa* from China including a new species redescribetion [sic]. *Journal of Hunan Normal University*, *Natural Sciences*, 26, 60-64.

Yang, Z. Z., Zhu, M. S. & Song, D. X. (2005). Two new species of the spider genus *Tmarus* Simon 1875 (Araneae: Thomisidae) from China. *Acta Arachnologica*, 54, 95-98.

Yin, C. M., Peng, X. J., Gong, L. S. & Kim, J. P. (2004). Two new species of the genus *Tmarus* (Araneae, Thomisidae) from China. *Korean Arachnology*, 20, 13-19.

Yin, C. M., Peng, X. J., Gong, L. S., Chen, Y. F. & Kim, J. P. (1997). Six new species of the genus *Pardosa* from China (Araneae: Lycosidae). *Korean Arachnology*, 13(2), 19-31.

Yin, C. M., Peng, X. J., Xie, L. P., Bao, Y. H. & Wang, J. F. (1997). Lycosids in China (Arachnida: Araneae). *Hunan Normal University Press*, 317 pp.

Yin, C. M., Peng, X. J., Yan, H. M., Bao, Y. H., Xu, X., Tang, G., Zhou, Q. S. & Liu, P. (2012). Fauna Hunan: Araneae in Hunan, China. *Hunan Science and Technology Press Changsha*, 1590 pp.

Yin, C. M., Wang, J. F., Zhu, M. S., Xie, L. P., Peng, X. J. & Bao, Y. H. (1997). Fauna Sinica: Arachnida: Araneae: Araneidae. *Science Press Beijing*, xiii, 460 pp.

Yin, C. M., Zhang, Y. J. & Bao, Y. H. (2003). Three new species of the genus *Oxyopes* from China (Araneae, Oxyopidae). *Acta Zootaxonomica Sinica*, 28, 629-633.

Zamani, A., Mirshamsi, O., Rashidi, P., Marusik, Y. M., Moradmand, M. & Bolzern, A. (2016). New data on the spider fauna of Iran (Arachnida: Aranei), part III. *Arthropoda Selecta*, 25(1), 99-114.

Zha, Z. W., Pham, D. S. & Li, S. Q. (2012). One new *Calommata* spider from Vietnam (Araneae, Atypidae). *Acta Zootaxonomica Sinica*, 37, 319-321.

Zhang, B. S., Zhang, F. & Zhang, Z. S. (2013a). Four new species of the genus *Pseudopoda* Jäger, 2000 (Araneae, Sparassidae) from Yunnan province, China. *Zootaxa*, 3702, 273-287. doi:10.11646/zootaxa.3702.3.5

Zhang, F., Zhang, B. S. & Zhang, Z. S. (2013b). New species of *Pseudopoda* Jäger, 2000 from southern China (Araneae, Sparassidae). *ZooKeys*, 361, 37-60. doi:10.3897/zookeys.361.6089

Zhang, J. X. & Zhu, M. S. (2005). Two new species of the spider genus *Oxyopes* (Araneae: Oxyopidae) from China. *Acta Arachnologica*, 53, 105-108.

Zhang, J. X., Yang, Z. Z. & Zhu, M. S. (2005). Two new species of the genus *Oxyopes* from China (Araneae: Oxyopidae). *Journal of Hebei University*, *Natural Science Edition*, 25, 75-78.

Zhang, J. X., Zhang, Y. Q. & Kim, J. P. (2005). A new species of the spider genus *Oxyopes* from Guangxi, China (Araneae: Oxyopidae). *Korean Arachnology*, 21, 1-5.

Zhao, J. Z. (1993). Spiders in the Cotton Fields in China. *Wuhan Publishing House Wuhan*, *China*, 552 pp.

Zhao, Q. Y. & Li, S. Q. (2014). A survey of linyphiid spiders from Xishuangbanna, Yunnan Province, China (Araneae, Linyphiidae). *ZooKeys*, 460, 1-181. doi:10.3897/zookeys.460.7799

Zhong, Y. & Liu, J. (2014). A new *Bhutaniella* species (Araneae: Sparassidae) from Taiwan Island, China. *Acta Arachnologica Sinica*, 23(2), 65-69. doi:10.3969/j.issn.1005-9628.2014.02.001

Zhu, C. D., Li, Z. S. & Sha, Y. H. (1986). Three new species of spiders of Linyphiidae from Qinghai Province, China (Araneae). *Acta Zootaxonomica Sinica*, 11, 264-269.

Zhu, M. S. & Zhang, B. S. (2011). Spider Fauna of Henan: Arachnida: Araneae. *Science Press Beijing*, xxii: 558 pp.

Zhu, M. S., Lian, W. G. & Ono, H. (2004). A new species of the genus *Lysiteles* (Araneae: Thomisidae) from Hainan Island, China. *Acta Arachnologica*, 53, 53-55.

Zhu, M. S., Song, D. X. & Zhang, J. X. (2003). Fauna Sinica: Invertebrata Vol. 35: Arachnida: Araneae: Tetragnathidae. *Science Press Beijing*, 418 pp.

Zhu, M. S., Zhang, J. X., Zhang, Z. S. & Chen, H. M. (2005). Arachnida: Araneae. In: Yang, M. F. & D. C. Jin (eds.) Insects from Dashahe Nature Reserve of Guizhou. *Guizhou People's Publishing House Guiyang*, pp. 490-555.
